# Immunopathology of Early Pregnancy

**DOI:** 10.1155/S1064744997000148

**Published:** 1997

**Authors:** Gérard Chaouat, Elisabeth Menu, Barbara Mognetti, Marléne Moussa, Véronique Cayol, Yakoute Mostefaoui, Sylvie Dubanchet, Philippe Khadel, Jean Luc Voluménie, Catherine Bertrand Rongiéres, Geneviéve Lapprée Delage

**Affiliations:** U 131 INSERM Hôpital Antoine Béclére Clamart 92140 France


Infectious Diseases in Obstetrics and Gynecology 5:73–92 (1997)
© 1997 Wiley-Liss, Inc.

### Immunopathology of Early Pregnancy

**Gérard Chaouat, Elisabeth Menu, Barbara Mognetti, Marléne Moussa, Véronique Cayol, Yakoute Mostefaoui, Sylvie Dubanchet, Philippe Khadel, Jean Luc Voluménie, Catherine Bertrand Rongiéres, and Geneviéve Lapprée Delage**

*INSERM, Hôpital Antoine Béclére, Clamart, France*

One of the important discoveries in reproduction is that the reproductive process uses for its own purpose lymphokines and cytokines which were initially defined as being of immunologic nature, first traced within the immune system. This is not very surprising, since most of these molecules are more signaling factors and communication molecules between cells of various lineages than molecules strictly restricted to the immune system, such as immunoglobulins (Ig) and T-cell receptors. The field has now evolved from the idea of a host–graft relationship to the concept of an integrated dialogue between the mother and the conceptus, more akin, for some, to a host–tumour relationship. Yet, in fact, the paradigm of acceptance and/or rejection of the foetus by its mother is still heavily embedded in the thinking of many reproductive immunologists.

This is not surprising, since the trend of research that led to modern reproductive immunology was initiated by Sir Peter Medawar's disquisition on "the riddle of the foetal allograft."[1] It is therefore not all disconcerting that, in 1997, one of Medawar's colleagues, Leslie Brent, in his recent book *A History of Transplantation Immunology*, still depicts the materno-fetal relationship as "Nature's (almost) perfect allograft,"[2] nor that pioneering work in the field is due to another collaborator of Leslie Brent and Peter Medawar, Rupert E. Billingham.[3]

However, the paradigms have evolved. As you will see in this brief presentation, there is still room for discussing, and re-envisaging, with the use of murine models and data in humans, the concept and mechanisms of "rejection" of the foetal allograft.

But the question of the tolerance of the conceptus has evolved. First, we have known for a long time that we *can* and *must* dissociate local and systemic events, but this idea has not yet fully penetrated the thinking of the general public.

It is possible to observe rejection of paternal strain tissues after preimmunisation of the mother without compromising pregnancy[4,5] (in fact, the babies born in such situations are in most circumstances heavier), and we have personally verified that.[6]

It ensues that systemic immune responses (which we do not want at all to negate, having taken a rather indisputable part in their discovery[7]) are, at best, a testimony of maternal recognition of the foeto-placental unit.

Indeed, pregnancy is perfectly possible in the absence of humoral (antibody) response.[8,9] Indeed, if tolerance to the foetus was alloantibody mediated, it would be an exception, because tolerance is MHC restricted, and the Nobel prize awarded to Zinkernagel and Doherty recognizes the consequences of that phenomenon. With rare exceptions, antibodies are *not* MHC restricted, and attempts to say that tolerance was antibody mediated should be in immunology history. This is not completely true, since there is not yet a satisfactory explanation of the enhancement phenomenon, possibly because of the controversy over IgG1/IgG2 in mice, which uselessly shifted the debate–enhancing antibodies in rats, *are* part cytotoxic.

Similarly, we do not negate, of course, the existence of systemic suppresser cells,[7] but pregnancy is perfectly viable in animals experimentally depleted of these.[10]

The repeated enunciation in France that pregnancy depends on an alloantibody and suppresser

Correspondence to: G. Chaouat: U 131 INSERM Hôpital Antoine Béclére, 92140 Clamart, France

Received 1 October 1997

Accepted 21 October 1997

T-cell-mediated immune response is simply outdated since around 1979–1981, and claims that unfortunately are expressed in mainstream immunology meetings and in print that "reproductive immunology has to teach the mainstream immunologists how the immune system works" as a "facilitation reaction" with "important role for enhancing antibodies . . . and suppresser cells" have disastrous effects:

- First, mainstream immunologists repeatedly dismiss, in a more and more irritated fashion, such preposterous advances, and, as such, would consider as a backlash the whole field as not serious;
- Second, those claims have led some clinicians to exacerbate the role of maternal antibodies, clouding unconsciously the issue of early pregnancy loss.
- Third, they distract from the main advances that have indeed been going on, leading to *molecular* understanding of the field.

For indeed, there has been progress, such as Tom Wegmann's seminal idea that possibly we should re-envisage our view of the immune system in pregnancy: instead of seeing the foetus as confronted by a threat of rejection by its mother—the "immunotrophic" concept postulated from a discovery made with the murine abortion model (the CBA x DBA/2 system),[11,12] we recognize that immune reaction could be in some cases *beneficial* for fetal survival.[13]

Then, it ensued the demonstration in vitro, then in vivo, that indeed T-cell derived cytokines were growth factors for the placental trophoblasts,[14] followed by in vivo demonstration of T-cell control of placental growth[15,16] and treatment of a murine abortion model (CBA x DBA/2) by purified or recombinant cytokines.[17]

In the very same period, David Clark had made the discovery that this precise model (CBA x DBA/2) was deficient in local, decidual associated suppresser factor,[18] and later on showed that this factor was molecularly related to TGF-b2.[19]

We are now faced with events occurring *mostly locally*, and with the concept of a cytokine network at the foeto-placental interface. The events are in fact *different* whether one looks at parturition (where there is good evidence for involvement of interleukin [IL]-1, IL-6, IL-8, and tumor necrosis factor [TNF][20–23,25]) or the established pregnancy which is indeed immunologically characterised by transient acceptance of relatively weakly immunogenic tumor grafts of parental strain origin in the first pregnancy, linked to a T-cell anergy state and suppresser T-cell mediated multi-pregnancy induced tolerance to paternal alloantigens.[26–29]

In the latter case, the close examination by polymerase chain reaction (PCR) of cytokine profiles shows that it is more complicated than initially thought, with variations throughout pregnancy of which the physiological significance is yet unclear,[30] and which we will not detail here in the human. Similarly, the profiles are not simple in mice.[131]

And we now come to the topic of this communication, early pregnancy. Nowhere is the understanding of the cytokine network more interesting, and nowhere has it led to more surprising discoveries, opposed to the paradigm of the acceptance of the foetal allograft, and so far away from the initial framework (which *was* indeed *once* useful) of the facilitation reaction.

Early implantation requires "inflammatory" molecules, the consequence, in part, of a quasi-inflammatory reaction locally, followed by an immediate return to non-inflammatory conditions. It is probable, from what we know in animal models, that each of these steps are *absolutely* required for further successful pregnancy. But before that, a word is required about the antigenicity of the embryo in mice and humans.

### AN ALLOGRAFT?

It is a tautology, a "Lapalissade" to say that to be alloantigenic, an embryo *must* express foreign tissues antigens. This is not totally true. Failure to express major histocompatibilty antigens has *another* counterpart. It *activates* cells which are what Charlie Loke calls the most primitive immune system,[31] the Natural Killer cells (NKs). That it was so is a relatively recent discovery, which owes a lot to the theory of the "missing self" originally proposed by Klas Karre.[32] Therefore, it ensues that the trophoblasts cannot theoretically be completely neutral, otherwise there would be an NK-mediated rejection reaction: the NKs express two sorts of receptors, which are not yet fully understood, albeit an impressive body of evidence is accumulating (see Immunological Reviews 158). Killer Activating Receptors (KARs), whose engagement, in a neoclassical fashion, triggers NK lytic pathways (and possibly others, such as cytokine secretion, especially when the Killer Inhibitory Receptors [KIRs] are engaged). The second, and most important, are KIRs. The KIRs are activated when engaged by the reconnaissance of the presence of MHC molecules (most often in a *given minor histocompatibility antigen context*), which explains "easily" some "oddities" which we published for the sake of honesty on the CBA xDBA/2 system and which were then rejected by proponents of the MHC-T-cell duo theory (proponents for whom we, incidentally, have the greatest respect), triggered so as to *inhibit* NK lytic pathway.

An added level of complexity for immunologists and clinicians is that while MHC is (relatively) conserved through evolution as a member of the super Ig family, KIRs and KARs are *not at all* related between mice and humans. *Why*?

Somehow, the trophoblast faces a dilemma: express MHC, which might trigger a classical T-cell-derived rejection reaction, or repress *completely* MHC, which will defuse the KIRs and trigger NK-mediated rejection.

This dilemma is partly solved in the human. The trophoblast expresses, as far as its extravillous component is concerned, a specific antigen, HLA-G, with no variations (or a few amino acids) from one individual to the other, but it is now known that it has some variability.[35] HLA-G has little peptide presenting capacity (*but* it does have it!), and, as a truncated molecule, it is not recognised as "polymorphic." (It has few antigenic disparities between individuals, if any, that can be "seen" by T-cell receptors, whereas it certainly inhibits NK function. Recent experiments by Strominger, Lopez Bottet et al, Ramensee, and Le Bouteiller confirm the earlier ones of Kovatts and Loke.[36,37,38])

The situation would thus seem simple in humans: the extravillous trophoblast is where interaction with decidual lymphocytes takes place and therefore where NK and NK-like cells accumulate and are defused. The lack of classical MHC molecules would render the trophoblast a non-target and a non-inductor of a classical T-cell-mediated response. In addition, there is no bioactive IL-2 in decidua, albeit an IL-2-like material has been traced.[39]

The situation is more complex than that, even for those who would like to make an exception in the case of primates: in humans, the expression of HLA-G is restricted to extravillous cytotrophoblasts, villous cyto-, and syncytiotrophoblasts that are uniformly MHC-negative, but a mirror situation is observed in baboons, and in the rhesus monkey, HLA-G homologue is a pseudo gene (although its function could be exerted by a yet uncharacterised protein).[40,41] So, this is already a snag.

Two further snags are to be discussed: First, how could the *totally* MHC-negative human syncytiotrophoblast, a fact not dismissed since its description by Faulk,[42,43] not trigger NK-mediated lysis? This is as yet unexplained by proponents of the HLA-G paradigm. Second, we now know that HLA-C is expressed by extravillous trophoblast.[37] It has a restricted polymorphism and interacts with NKs, but the theory must somehow be twisted to make it act as purported for the monomorphic HLA-G, for despite a certain paucity of reports, there are T-cell-mediated responses to HLA-C.

Finally, in *all* species other than the primates, there is expression of classical, *polymorphic* class I molecules, generally *precisely* on the layers of the placenta that confront the maternal immune system (44,45,46). In mice K, D, and L are expressed on the spongiotrophoblast, perfectly accessible to antibodies and cells. The same is true in horses for equine leukocyte antigens, in pigs for swine leukocyte antigens, and, according to Twink Allen, in elephants.

There are three alternative hypotheses for the problem:

- One is that man is man, or, as Peter Johnson wrote (The Immunologist, 1996:4;p172), "differences in placentation between humans, rodents, and other species can make direct comparisons largely meaningless."
- The second one is, as Leslie Brent says when discovering the status of HLA-G in other species, that HLA-G suppresses as would any proper MHC-ligand in the proper cells (that is, by the sort of experiments in which the missing self was discovered to be true) and that HLA-G has likely *another* function, since there is no equivalent in mice, etc, that warrants its evolutionary appearance (a specific peptide or hormone intracellular carrier, for example, yet uncovered).

- The third one is that *there are* in other species *functional* equivalents of HLA-G, which *could* reside in the already discovered monomorphic or poorly polymorphic MHC class I molecules.

None of these theories, incidentally, is mutually exclusive.

However, for class II (I-a in mice, HLA-DR in humans), the situation is more simple. In no species do the trophoblast layers express class II alloantigens, and expression of class II on trophoblasts induced by azacytidine results in regular abortion in mice.[47]

Why? The question is unsettled, since the mechanisms have not been explored, but one should recall here in the same vein that expression of class I in mice is restricted to spongiotrophoblast, with no expression being found on labyrinth. Indeed, derepression of class I expression in the labyrinth is found in interferon $\gamma$ induced abortion, but one does not know if it is a consequence or a cause of abortion.[48] So, we are in quicksand about the "allograft status of the embryo."

As far as the preimplantation embryo is concerned, it is simpler to say *that there is no class I* expression (nor class II) whatsoever on the gametes, blastocysts, extracellular cell mass or ectoplacental cell cone (EPC) in *any* species, including, when studies could be done, humans.[49] This is somehow troublesome for some aspects of the recent HLA-G theories: the negative EPC cells certainly are facing a very important NK accumulation.[50] And yet, the EPC resists perfectly well NK-mediated lysis. Conversely, aborted embryos in many species at that early stage display MHC expression on EPC cells.

The solution may be simple: at that stage, the cells of the EPC are *intrinsically* resistant to cell-mediated lysis,[51–53] most probably because cell-mediated death involves target cell participation and the pathways of apoptosis. Like other cells secreting high amounts of steroids, trophoblasts are resistant to steroid-induced apoptosis and thus to cell-mediated death. However, they are sensitive to LAKCs mediated cell death,[54] but for that we will see that there is in addition a lot of immunosuppressive material in the vicinity, and that abortion might *not* be mediated by trophoblast death. Such an immunosuppression takes place after implantation, which on the contrary calls for inflammation, and is a Th1 response.

### INFLAMMATION AND IMPLANTATION

In France, the early stages of pregnancy are difficult to study in humans, despite advances in medically assisted reproduction. The Loi Huriet requests full informed consent before taking a biopsy sample, and in the very "sensitive" area of reproductive tract organs, this is quite a deterrent, as opposed to the ease with which blood samples are obtained.

In the implantation period, where some events are very transiently associated with blastocyst adhesion and initial invasion of uterine walls, it is, of course, inconceivable and unlawful to deliberately transfer an in vitro fertilisation (IVF) for the sole purpose of aspiration or, even worse, (total) removal of uterine tissues for immunological investigations. As stated, animal models are not necessarily fully pertinent, and thus Y.W. Loke said at the 15th World Congress on Fertility and Sterility in Montpellier that the only valid model for human pregnancy is "the human species itself."

But, this does not solve fully the problem of the periimplantation period. Macaques are not fully relevant, because of antigenic disparities, like baboons, at the trophoblast level with human situation, and chimpanzees are an endangered and protected species of extremely limited availability. For these reasons, in vitro alternative models have been developed, which may or may not be totally relevant, complemented by studies in animals, mostly in mice.

### Animal Studies

The studies performed in animals have revealed a salient and totally unexpected aspect contrasting with the established pregnancy: the preimplantation uterus undergoes an "inflammatory-like" reaction, with transient influx, after mating in mice and rats, there of lymphocytes and macrophages, and seminal fluid is required, since this is not seen in females with fallopian tubal ligation or in vasectomised males.[55–57] As expected, an increased secretion of IL-1 $\alpha$, IL-1 $\beta$, TNF $\alpha$, IL-6, and mRNA is detected in the uterus, paralleled by the pregnancy-associated continuous rise in production of CSF-1 which starts by then.[58,59] Except for CSF-1, those inflammatory cytokines return to basal levels by days 3 and 4, but CSF-1 secretion continues to increase throughout pregnancy.[58,59] In rats, local injection of the PAF-acether antagonist BN 50081 completely prevents implantation, whereas the untreated contralateral horn is fully receptive.[55,56]

Three cytokines are, in our opinion, crucial, as established in mutant or "knock-out" animals: CSFs, IL-1, and LIF.

### CSFs (CSF-1, S-CSF, GM-CSF)

CSF-1 deficient oSP (osteopetrotic, or op/op) mutant mice have a profound pregnancy defect, and males have also reproductive functions. CSF-1 receptor, c-fms, is expressed after the 2–4 cell stage until the EPC outgrowth and later the spongiotrophoblast and is also found at very high levels in the maternal then the primary decidual zone and finally in the sole decidua basalis.[62] S-CSF and its receptor (c-kit, which maps in the dominant white spotting W locus in the mouse), is also important, since mutations in the W locus t result in sterility in mice, often associated with abnormal embryonic development.[62]

GM-CSF was the first cytokine with IL-3 involved in the immunotrophic theory[13–16] and is found in the preimplantation uterus. Tartakowsky has shown that culture of CBA × DBA/2 embryos (a murine abortion model[11,12]) in GM-CSF- or CSF-1-containing medium partly corrects the deficient implantation rate seen when transferring these embryos in CBA /J mothers.[63] GM-CSF receptor is expressed in the EPC and later spongiotrophoblast cells and in the early implantation uterus.[64,65] Recently, in an elegant series of experiments, Robertson implied the inflammatory response and more peculiarly the GM-CSF. In agreement with studies conducted to prove the immunotrophic theory,[15,16,66] she found that GM-CSF-deficient mice have smaller litter sizes and placental weights than controls and implies GM-CSF and components in the semen ejaculate in hyporesponsiveness to paternal alloantigens.[67]

### IL-1

IL-1 might play a mandatory role in pregnancy. According to Simon and Polan,[68] IL-1 receptor antagonist completely prevents successful implantation in mice. However, Colin Stewart et al. (Stewart, personal communication and answers at many meetings) have examined at the Roche Institute for Molecular Biology IL-1 deficient mice with perfectly normal reproductive function, nor did we obtain implantation failure in mice by in vivo injection of neutralising anti-IL-1 antiserum.

### LIF

By gene knock-out technology, Stewart has elegantly shown that maternal production of LIF is mandatory for successful implantation: LIF-deficient mice, obtained by "gene knockout" are fertile, *but sterile.* LIF-/LIF- embryos implant in normal CD1 recipient mice, but LIF +/LIF + embryos do not implant in LIF-/LIF- mice. The defect is corrected by recombinant HILDA LIF via an osmotic pump.[69]

### Human Studies

### IL-1

IL-1 α, β expression is found in human endometrial epithelium throughout the menstrual cycle, reaching a peak in the late luteal phase.[70,71] Immunohistochemistry shows that IL-1 receptor antagonist in human endometrial cells is at higher levels during the follicular phase than during the early and mid luteal phases.[72] IL-1 is also found in trophoblasts and IL-1 receptor type I on syncytiotrophoblasts, suggesting an autocrine and paracrine role of IL-1 in human implantation.[73] Indeed, it has been reported that IL-1 is a modulator of the decidualisation itself,[74] and it is also controlling hCG production by human trophoblasts in culture.[75] The production and role of IL-1 by early human embryos is controversial; some authors made it a predictor of successful implantation in humans,[76,77] while others did not detect it at all.[34,78] Differences in culture media, conditions, etc. obviously could be invoked. In a co-culture of endometrial epithelial cells and or stromal cells with embryos, IL-1, IL-1 receptor antagonist, and IL-1 receptor type 1 were found in about 56% of cells from embryos cultured with epithelial cells but not stromal cells, apparently correlated with pregnancy success.[79]

### LIF

LIF is present in human endometrium, with consensus for its expression in the second part of the cycle, and during pregnancy, this in variance with mice.[80–82,84] Optimal uterine secretion coincides with the implantation window and expression of

TABLE 1. Endometrial HILDA/LIF production index in fertile women

| HILDA/LIF production index[a] | Cycle day | Age (years) | Obstetrical status: Number of successful pregnancies | Obstetrical status: Last pregnancy (years) |
|---|---|---|---|---|
| 1.05 | 20 | 50 | 2 | 18 |
| 1.66 | 6 | 36 | 2 | 4 |
| 1.94 | 14 | 41 | 2 | 20 |
| 2.21 | 10 | 37 | 4 | 5 weeks |
| 2.22 | 20 | 41 | 2 | 1 |
| 2.28 | 7 | 32 | 2 | 9 |
| 2.35 | 22 | 34 | 2 | 10 |
| 3.16 | 10 | 36 | 1 | 12 |
| 3.27 | 18 | 32 | 1 | 1 |
| 3.39 | 10 | 41 | 5 | 14 |
| 3.85 | 19 | 35 | 2[b] | 6 weeks |
| 4.06 | 19 | 36 | 2 | 12 |
| 4.51 | 22 | 32 | 2 | 4 |
| 4.57 | 37 | 30 | 1 | 5 |
| 6.48 | 11 | 38 | 2 | 8 |
| 9.95 | 24 | 41 | 1[b] | 1 |

[a]Median = 3.22.
[b]Plus one pregnancy loss before successful pregnancy.
From: Delage G, Moreau J-F, Taupin J-L, et al.: In vitro endometrial secretion of human interleukin for DA cells/leukaemia inhibitory factor by explant cultures from fertile and infertile women. Hum Reprod 10:2483–2488, 1995.

LIF receptor by human embryos.[85,86] We have found women with quantitative defects in LIF production;[87,88] we have observed such a deficiency in women with successful IVF but confirmed sterility.[87,88] We have now confirmed those data, obtained in a culture system, by both immunohistochemistry, using polyclonal antibodies, and evaluation of LIF production by isolated uterine epithelial cells.

Tables 1, 2a, 2b, and 3 show typical LIF production indexes in fertile and infertile women, as defined, in an in vitro assay system, by the index of LIF production by explant cultures in culture supernatants from Day 5 and Day 1. It is interesting to note that in women whose most recent reproductive event was a pregnancy loss, there are a great number with a low LIF production index (Table 3), the indexes being *very* low for women with recurrent implantation failure or recurrent unexplained sterility (Tables 4 and 2b). The existence of a low producer of LIF has been confirmed ex vitro by uterine flushing and ELISA assays.[89]

As stated above, we wanted to have a totally objective measure of LIF production in situ by the human endometrium. We therefore set up the technique of selective separation digestion of glandular and stromal endometrium, after a Cornier of Frydman pipette biopsy sample, and using polyclonal antibodies raised in cooperation with J Martal (INRA, France), E. Boosmans (Eurogenetics, Belgium), and J F. Moreau and J L. Taupin (CNRS, Bordeaux). Data show that there is always a specific staining in the glandular cells, though contaminating cells of this origin can account for the low number of positive cells in the "stromal" compartment, which is in fact "negatively selected." One observes, as a result of trypsin digestion, that dead cells are already permeated, thus accounting for the positive staining seen in the non-permeated populations. We will give two experiments as examples, values being expressed as mean fluorescence intensity (which cannot be compared from one experiment to another, of course, but in the future we will always refer to the positive clone 8 values). On May 16, 1997, we found a mean value of 52.75 in an infertile woman, vs. 112.26 and 137.43 in two fertile ones, and on April 10, 1997, 108.75 in the fertile woman, vs. greater than 90.39 in infertile woman number 1 and greater than 37.27 in infertile woman number 2. On May 20, 1997, we similarly found 401.11 in the fertile woman, vs. greater than 158.25 in the infertile woman. Though this technique is somewhat limited by the restricted availability of the rabbit antiserum used (not all the rabbit anti sera were useable for FACS analysis, and both an excellent neutralising goat antiserum that we raised as well as commercial monoclonals proved unreliable in that respect), we

TABLE 2A. Endometrial HILDA/LIF production index in infertile women with recurrent implantation failure

| HILDA/LIF production index[a] | Cycle day | Age (years) | Number of IVF with embryos | Number of pregnancies | Last pregnancy (years) |
|---|---|---|---|---|---|
| 0.96 | 10 | 34 | 3 | 1 (EUP) | 15 |
| 0.97 | 5 | 29 | 20 | 3 (2 PL, 1 EUP) | 5 |
| 0.97 | 21 | 34 | 8 | 2 (1 EPT, 1 EUP) | 4 |
| 1.01 | 6 | 34 | 2[b] | 1 (PL) | 9 |
| 1.22 | 15 | 35 | 3 | 0 | |
| 1.41 | 10 | 39 | 1[b] | 0 | |
| 1.49 | 18 | 33 | 2 | 0 | |
| 1.68 | 9 | 36 | 6 | 0 | |
| 1.69 | 21 | 36 | 2 | 0 | |
| 1.70 | 7 | 34 | 4 | 0 | |
| 1.74 | 14 | 33 | 4 | 1 (EUP) | 10 |
| 2.45 | 18 | 38 | 6 | 0 | |

[a]Median = 1.45.
[b]Plus GIFT.
EUP = extrauterine pregnancy; PL = pregnancy loss; EPT = elective pregnancy termination.
From: Delage G, Moreau J-F, Taupin J-L, et al.: In vitro endometrial secretion of human interleukin for DA cells/leukaemia inhibitory factor by explant cultures from fertile and infertile women. Hum Reprod 10:2483–2488, 1995.

TABLE 2B. Endometrial HILDA/LIF production index in infertile women with unexplained primary sterility

| HILDA/LIF production index[a] | Cycle day | Age (years) |
|---|---|---|
| 1.22 | 12 | 41 |
| 1.28 | 6 | 30 |
| 1.39 | 8 | 30 |
| 1.48 | 6 | 33 |
| 2.70 | 13 | 34 |

[a]Median = 1.39.
From: Delage G, Moreau J-F, Taupin J-L, et al.: In vitro endometrial secretion of human interleukin for DA cells/leukaemia inhibitory factor by explant cultures from fertile and infertile women. Hum Reprod 10:2483–2488, 1995.

believe we have an objective assay that can complement immunohistochemistry, analysis on cytofuge smears, and in vitro production in explant culture.

It is important, in this context, to emphasize that recombinant human HILDA LIF is available, and women could benefit from substitutive therapy. While we feel confident enough to go forward with such a procedure, the clinicians would prefer we first correct a defect outside pregnancy before going further on.

This leads to questions about the mechanisms of action of LIF and, indeed, the inflammatory response. It is our working hypothesis that the local inflammation is required for optimal expression on placental trophoblast and uterine decidual cells of adhesion molecules, which are necessary for implantation to occur, such as trophoblast laminin and fibronectin receptors, and specifically the α 1 β 1, α 5 β 1, α 6 β 1, and α 6 β 4 integrin heterodimers, and expression of their receptor molecules (laminin and fibronectin) in the periimplantation as well as first trimester uterus with specific trophoblast down-regulation of expression of the β 4 integrin and up-regulation of the β 1, α 5, α 1 subunits during the invasion process.[91–93]

We believe that an important part of early pregnancy loss is due to abnormal processes of this early reaction. It is noticeable that dysregulations are already known, such as in preeclampsia, where trophoblasts do not down-regulate β 4, nor is α 1 up-regulated.[94] In addition, the effects of cytokines on the matrix degrading protease are also to be encompassed in that respect.[31]

### *TNF*

There is early and temporary expression of relatively high levels of TNF by invading extravillous trophoblasts[95] which could, as seen in other systems, up-regulate adhesion molecules and selectin.[96] This inflammatory reaction, however, has to be then very quickly down-regulated, since during the early post implantation phase it is abortogenic. There are many other cytokines that we will not discuss here, for sake of space, but we cannot *not* mention here CSF-1 and its receptor, whose programme is very parallel to what is seen in rodents and which has a similar role,[97–101] mainly as placental trophoblast growth factor acts as the other immunotrophic cytokine, IL-3[102] In this commu-

TABLE 3. Endometrial HILDA/LIF production index in women whose last reproductive event was a pregnancy loss

| HILDA/LIF production index[a] | Cycle day | Age (years) | Number of successful pregnancies | Last successful pregnancy (years) | Number of pregnancy losses | Last pregnancy loss (years) |
|---|---|---|---|---|---|---|
| 0.98 | 15 | 38 | 0 | | 1 | 12 |
| 1.12 | 15 | 31 | 0 | | 1 | 4 |
| 1.20 | 19 | 34 | 2 | 11 | 2 | 3 and 2 |
| 1.79 | 9 | 35 | 0 | | 2 | 2 |
| 1.86 | 6 | 33 | 0 | | 1 | 16 |
| 1.89 | 20 | 34 | 0 | | 4 | 1 |
| 1.98 | 13 | 36 | 3 | 3 | 1 | 1 |
| 2.33 | 19 | 38 | 2 | 6 | 1 | 4 |
| 2.38 | 19 | 41 | 2 | 13 | 1 | 1 |
| 2.43 | 22 | 32 | 0 | | 2 | 3 |
| 2.45 | 12 | 35 | 0 | | >20 | 0.5 |
| 2.79 | 14 | 41 | 1 | 5 | 1 | 3 |

[a]Median = 1.94.

From: Delage G, Moreau J-F, Taupin J-L, et al.: In vitro endometrial secretion of human interleukin for DA cells/leukaemia inhibitory factor by explant cultures from fertile and infertile women. Hum Reprod 10:2483–2488, 1995.

TABLE 4A. Endometrial HILDA/LIF production index in infertile women with recurrent implantation failure

| HILDA/LIF production index[a] | Cycle day | Age (years) | Number of IVF with embryos | Number of pregnancies | Last pregnancy (years) |
|---|---|---|---|---|---|
| 0.96 | 10 | 34 | 3 | 1 (EUP) | 15 |
| 0.97 | 5 | 29 | 20 | 3 (2 PL, 1 EUP) | 5 |
| 0.97 | 21 | 34 | 8 | 2 (1 EPT, 1 EUP) | 4 |
| 1.01 | 6 | 34 | 2[b] | 1 (PL) | 9 |
| 1.22 | 15 | 35 | 3 | 0 | |
| 1.41 | 10 | 39 | 1[b] | 0 | |
| 1.49 | 18 | 33 | 2 | 0 | |
| 1.68 | 9 | 36 | 6 | 0 | |
| 1.69 | 21 | 36 | 2 | 0 | |
| 1.70 | 7 | 34 | 4 | 0 | |
| 1.74 | 14 | 33 | 4 | 1 (EUP) | 10 |
| 2.45 | 18 | 38 | 6 | 0 | |

[a]Median = 1.45.
[b]Plus GIFT.

EUP = extrauterine pregnancy; PL = pregnancy loss; EPT = elective pregnancy termination.

From: Delage G, Moreau J-F, Taupin J-L, et al.: In vitro endometrial secretion of human interleukin for DA cells/leukaemia inhibitory factor by explant cultures from fertile and infertile women. Hum Reprod 10:2483–2488, 1995.

nication, however, we would like to mention tau and placental interferons.

### Placental Interferons

Corpus luteum maintenance in ovine does not depend on non-chorionic gonadotropin, but on trophoblastin whose trophoblast secretion occurs in the periimplantation, also named ovine trophoblast protein (oTP). It has pleiotropic activities.[103,104] It exerts a wide variety of effects, such as local antiviral properties, preparation of the uterus for optimal implantation by promoting 2 5 A synthetase activity, and local cytostatic properties, making it an ideal candidate for early immune suppression. It is likely to be involved in control of the Th1/Th2 balance.[105–108] It is in fact an interferon of the new

**TABLE 4B. Endometrial HILDA/LIF production index in infertile women with unexplained primary sterility**

| HILDA/LIF production index[a] | Cycle day | Age (years) |
|---|---|---|
| 1.22 | 12 | 41 |
| 1.28 | 6 | 30 |
| 1.39 | 8 | 30 |
| 1.48 | 6 | 33 |
| 2.70 | 13 | 34 |

[a]Median = 1.39.
From: Delage G, Moreau J-F, Taupin J-L, et al.: In vitro endometrial secretion of human interleukin for DA cells/leukaemia inhibitory factor by explant cultures from fertile and infertile women. Hum Reprod 10:2483–2488, 1995.

tau interferon family. When such trophoblastins exist, they are constitutively secreted at very high doses locally by periimplantation trophoblast only during the periimplantation phase. It is non abortogenic (of course, *it maintains* pregnancy) and non toxic/cytostatic for trophoblast, which secretes it, while ? interferon at high doses is an abortifacient in a variety of animal models. The quest is still ongoing for human equivalents, but not conclusively, despite suggestive evidence[109], so one speculates that its functions could in fact be exerted by omega interferons.

### Th1/Th2 BALANCE AND LOCAL IMMUNOSUPPRESSION

As soon as one completes the implantation period, in humans and in mice, an abnormal Th1 reaction to trophoblast causes early pregnancy loss, recurrent spontaneous abortion, and infertility. Several mechanisms are involved. For example, the HELLP syndrome (hemolysis, elevated liver function tests, and low platelet counts) is linked to an elevated IL-12 serum level and possibly an enhanced IL-2 level in the serum[110,111] of unclear origin, since despite recent molecular evidence for the contrary[112], no IL-2 is assumed to reside in decidua and placenta. This is especially important, since in a variety of animal systems, activated NKs (by IL-2 or IL-4, e. g, LAKCS), are abortifacient, and indeed LAKCS can lyse trophoblast cells in vitro,[54] but not normal NKs or CTls. Thus, there has been a quest as indeed "built-in" in the "allograft model" for local immunosuppression for the mechanisms of early pregnancy loss and its control.

The Th1 cytokines, be they IL-2, interferon γ, or TNF, are abortifacient in vivo, the latter at *any* stage of pregnancy, in abortion-prone and non-abortion-prone systems[17,113,114,116,117] Interferon γ and TNF act in synergy. Their control is exerted by a variety of non-cytokinic mediators (prostaglandins, 1-25 OH dihydroxyl cholecalciferol in the decidua, placental low MW suppresser factors), as well as by suppressive cytokines such as TJ6 (the "pregnancy-associated cytokine") which has been cloned initially in mice. Monoclonals against TJ6 are abortifacient at early stages, and deficient expression of TJ6 is reported to be associated with pregnancy loss in human, especially if the defect is on CD 19- and CD 56-positive peripheral and decidual lymphocytes. Such a defect could be predictive of risk of pregnancy loss.[118–121] Another regulator which is, like TJ6, progesterone dependent is the progesterone induced blocking factor (PIBF), secreted by activated T lymphocytes.[122] PIBF alters the Th1/Th2 balance towards Th2 and corrects activators of NKK (poly IC) and natural (CBA × DBA/2) induced abortion.[123–125] Its expression by maternal lymphocytes has been shown to correlate with (perhaps even require) implantation.[126] The decidua-associated transforming growth factor β-2 analogue[19] is secreted by uterine non-T, non-B cells in mice and decidual transforming growth factor β-2 secreting cells in humans.[126] It is a potent immunosuppressive factor, and it has been reported that its production was defective in women with repeated miscarriages.[126] Finally IL-10 is clearly important, since in animal models pregnancy is very obviously a Th2 phenomenon.[127] In humans, it is secreted by placental trophoblasts and choriocarcinoma,[128,129] and, interestingly, its secretion is enhanced by GM-CSF. IL-10 is also produced by isolated decidual cells.[130] In mice, its secretion is more controversial.[131,132] But, in murine models, IL-10 with IL-3 suppress local action of IL-2 and gamma interferon, biasing the maternal immune response towards a Th2 profile. Deneys (UCL, Bruxelles, Belgium) has shown that in pregnant women, TNF was down-regulated and IL-4 up-regulated when measured in the serum, a pattern not seen in recurrent aborters (Deneys, personal communication). Further proof of the role of IL-10 is the effect observed in infections. Leishmania biases the responses towards Th1, and there is less IL-10 but more pregnancy failure.[133,134]

### EFFECTOR MECHANISMS

What are the exact mechanisms of early pregnancy loss? It is clear that interferon and gamma interferon are involved, very often as a consequence of either abnormal maternal recognition of the conceptus/semen, or local, undetected "*a très bas bruit*" infection, with peculiar insistence on mycoplasma.[17,108,133–136] The data of Hill strongly suggest it is also the case in humans, e.g., Th1 immunity causes recurrent abortions and sterility, with emphasis on gamma interferon.[137,138] But what are the exact mechanisms?

First, the cells and the "immunological events" need to be reconsidered, even for immunotrophism; interest is leaning now toward NKs rather than T cells. Anne Croy has shown that placentae of NK-deficient mice are grossly hypotrophic, leading to premature foetal death,[139] shifting the "immunotrophic" paradigm from T cells to NK cells. Thus, the production of immunotrophic cytokines might be in fact *mostly* (but not exclusively) under direct control of NK cells more than T cells, and "allorecognition" of pregnancy might, in fact, be exerted by these NKs rather than in a classical fashion by T cells. In consequence, a hitherto little suspected role could be envisaged for HLA-G: the control of the production of immunotrophic cytokines and not solely the local defusing of NK mediated cytolytic functions (a role that was forecasted by Y W Loke[31]).

In the CBA x DBA/2 model of natural immune abortion, together with the poly IC 12 U model, (an NK activator),[142] we explored the role of NKs in cooperation with P. Kourilsky.[140,143,144] Anti MHC H-2d (BALB/c) alloimmunisation prevents the effect of Poly IC or Poly I Poly C12 U as well as CBA x DBA/2 fetal loss. *But, not all* selective alloimmunisation prevents the CBA x DBA/2 foetal loss, and the genetic patterns of the effective immunising splenocytes (MHC restricted, minor loci dependent), albeit apparently representing a single trait,[12,141] are so odd to comprehend in terms of "classical" T-cell recognition (BALB/c works in CBA xDBA/2, B10,D2 does *not–both* are H-2d) that some T-cell and MHC proponents preferred to dismiss the data as non reproducible (which they were not, being reproduced at present by more than 20 labs, with significant developments) or (we quote) "absolute bullshit."

But, we then demonstrated that immunisation with Kd or Ld transfected L cells prevented resorptions and used that system to study the effect of amino acid mutations on the border of the pocket of the MHC molecule in that system. These were shown to affect recognition by maternal cells promoting optimal foetal survival when immunisation was performed using $H\text{-}2^d$ mutant transfected L cells. We then used various mutations that were artificially induced and localized on the border of the molecule's groove or in the peptide binding site. Some H-2d mutations on the helixes are recognized as foreign to H-2d and afford foetal protection against Poly IC. With P. Kourilsky, we wondered whether NK recognition of discrete determinants on the MHC molecule was not involved and, with help of Sylvie Delassus, Jean Pierre Abastado, Claude Roth, and Jos Even, we immunised CBA/J with L cells transfected with H-2d molecules mutant on positions 65, 69, which do not affect peptide presentation and 114 as a control. These protected against CBA x DBA/2 fetal loss, and, more important, enhanced IL-10 production in the supernatant of placental and decidual explants as already described using a commercial ELISA of high sensitivity, while treatment with asialo GM1 abolishes this effect.

At this stage, we propose the following working hypothesis: the NK cell repertoire is heteroclitic and consists of the recognition of selected altered domains of MHC molecules, whether that alteration results from mutations or allosteric conformational changes induced by (in the context of) background genes. Such a recognition either switches lytic/cytostatic pathway (macrophage activation, NK activation, TNF secretion, and balance of the CD4 system towards a Th1 profile) to IL-4 and IL-12 secretion by the NKs or pushes the system towards IL-10 secretion, and secretion by NKs or under the influence of NKs of the so-called "immunotrophic" cytokines. Thus, the concept would integrate *both* immunotrophism and the Th1/Th2 balance.

As far as the effector mechanisms are involved, it is already well known that TNF and interferon are causative and act in synergy,[108,145,146] but other mediators have been implied, such as nitric oxide released by activated macrophages.[147–150] There is

TABLE 5. Role of asialo GM1 + NK cells and macrophages in abortions

| Expt | Day 6.5 treatment | Day 7.5 treatment | Day 13.5 assay | N[a] resorptions/total | % abortions |
|---|---|---|---|---|---|
| 1 | PBS | PBS | 8 | 23/56 | 41% |
| | PBS | 1000 υ TNF-α | 8 | 43/60 | 72%[b] |
| | PBS | 2000 υ TNF-α | 8 | 57/64 | 89%[c] |
| | anti-asialo GM1 | PBS | 8 | 10/59 | 19%[d] |
| | anti-asialo GM1 | 1000 υ TNF-α | 8 | 12/63 | 16%[e] |
| | anti-asialo GM1 | 2000 υ TNF-α | 8 | 12/55 | 22%[e] |
| 2 | PBS | PBS | 16[f] | 43/101 | 43% |
| | PBS | 1000 υ γ-IFN[g] | 16 | 79/93 | 85%[g] |
| | PBS | 1000 υ γ-IFN + TNF-α[h] | 16 | 74/89 | 83%[h] |
| | anti-asialo GM1 | PBS | 16 | 11/71 | 15%[d] |
| | anti-asialo GM1 | 1000 υ γ-IFN | 16 | 12/98 | 12%[e] |
| | anti-asialo GM1 | 1000 υ γ-IFN + TNF-α[h] | 16 | 89/104 | 86%[h] |
| 3. ctrl[i] | | PBS | 8 | 36/88 | 41% |
| ctrl | | γ-IFN + TNF-α[j] | 8 | 65/80 | 81%[j] |
| $SiO_2$[k] | | PBS | 8 | 14/55 | 25%[k] |
| $SiO_2$ | | γ-IFN + TNF-α[j] | 8 | 52/65 | 80%[j] |

[a]N represents number of pregnant mice per group.
[b]Significant increase in abortion rate, $P < 0.005$ by $\chi^2$.
[c]Significant increase in abortion rate compared to PBS control, $P < 0.005$ by $\chi^2$; significant difference compared to lower dose of TNF-α, $P < 0.05$.
[d]Significant reduction in abortion rate by anti-asialo GM1 antibody compared to PBS control, $P < 0.005$ by $\chi^2$.
[e]No significant boosting of abortion rate compared to PBS injected anti-asialo GM1-treated group.
[f]Result from two independent experiments giving same result have been pooled.
[g]γ-interferon (γ-IFN) significantly boosted abortion rate, $P < 0.005$ by $\chi^2$.
[h]TNF-α was given at 1000 υ and 2000 υ in separate experiments with γ-IFN and gave similar results; the data have been pooled for ease of presentation. The abortion rate was significantly boosted, $P < 0.005$ by $\chi^2$.
[i]Untreated CBA/J female mice mated to DBA/2 males.
[j]1000 υ γ-IFN + 2000 υ TNF-α significantly boosted abortion rate, $P < 0.005$ by $\chi^2$.
[k]CBA/J mice injected twice a week for 4 weeks with 100 mg/kg silicon dioxide before mating significantly reduced abortion rate, $P < 0.05$ by $\chi^2$.

no doubt that asialo GM1+ cells or cells of the NK lineage are involved, since their transfer, once activated, causes abortion, and, since they do accumulate at the site of embryo resorption, their modulation positively (Poly IC activation) or negatively (anti-asialo GM1 treatment) influences parallel resorption rates.[151–154] We have studied this problem with David Clark, taking into account the fact that TNF and gamma interferon are by themselves abortifacients, and performing cell deletion to learn the cellular mechanism triggered.[155]

In Table 5, intraperitoneal injection of TNF-α enhanced CBA xDBA/2 resorptions, but anti-asialo GM1 antibody both decreased the background rate and prevented the action of TNF-α. When we added γ-interferon, it had as expected an abortifacient effect, but that was not seen in NK-cell-depleted mice, suggesting that the Baines model[149,150] (-interferon ( macrophages ( activated to produce NO ( embryo death was not correct. However, when γ interferon and TNF-α were administered together, more than 80% of the implanted embryos aborted. This confirmed the already observed synergy/codependence and suggested that in fact it might be crucial. Indeed, NK cell depletion suggested that TNF-α could not find enough NK-derived γ-interferon, while γ-interferon alone fails because macrophages which depend on NK-cell-derived γ-interferon have and thus did not produce TNF-α, and the intraperitoneally injected cytokine does not stimulate TNF-α production quickly enough.

The action of NKs or macrophage has been attributed to a direct killing of trophoblast. In silica treated macrophage-depleted mice, the abortion rate was reduced, *but* this treatment was ineffective in TNF-α plus γ-interferon treated animals, whose macrophage depletion was checked by the anti F4/80+ MoAb. Ovarian failure could have happened after silica, but this was ruled out since it should have caused abortions in 100% of cases, and the results were unaltered hormone replacement therapy treated animals.

The most logical target appeared to be the maternal uterine vascular endothelial cell, since the cytokines stimulate surface expression of procoagulant (flg/2-prothrombinase, which is distinct from tissue factor) and clotting. Table 6 shows that an-

TABLE 6. Antibody to flg/2 prothombinase prevents abortions in DBA/2-mated CBA/J mice

| Pretreatment group | Day 7.5 treatment | Day 13.5 assay | N resorptions/total | % abortion |
|---|---|---|---|---|
| Control Rabbit IgG | nil | 8 | 21/56[a] | 38% |
| Control Rabbit IgG | γ-IFN + TNF-α[b] | 8 | 48/55 | 87%[c] |
| Rabbit IgG anti-flg/2 | nil | 9 | 3/66 | 4.5%[d] |
| Rabbit IgG anti-flg/2 | γ-IFN + TNF-α[b] | 9 | 9/68 | 13%[e] |

[a]Results from two independent experiments which gave the same result.
[b]1000 υ γ-IFN and 2000 υ TNF-α was injected intraperitoneally.
[c]Significant increase in abortion rate $P < 0.001$ compared to no cytokine control group. Fisher's Exact test.
[d]Significant reduction in spontaneous abortion rate $P < 0.001$ compared to no cytokine control group, Fisher's Exact test.
[e]Significant reduction in abortion rate $P < 0.001$ compared to cytokine-treated controls, Fisher's Exact test. No significant difference compared to anti-flg/2-treated mice which did not receive an injection of cytokines.

tibody to flg/2 prothrombinase reduced the background rate of abortion to 4% and prevented the effects of TNF-α plus γ-interferon. Since granulocytes are observed in resorption sites and lyse of TNF-α plus γ-interferon-activated endothelial cells, we tested the injection of pregnant mice with a monoclonal anti-granulocyte antibody which is known to block granulocyte-mediated tumor rejection in vivo.[156] Anti-granulocyte antibody partially reduced the spontaneous abortion rate and significantly abrogated the effect of TNF-α plus γ-interferon (as control, see effects of rIL-10) (Table 7). Thus, vasculitis, rather than a direct cytotoxic action on fetal trophoblast, leads to abortion.

In this context, and in keeping with the aforementioned rôle of NK cells in cytokine production, concerning protection against abortion, TGF-β2-producing γδ T cells in the uterine lining appear to be important.[157,158] These cells are producing TGF-β2 and IL-10, albeit, by in situ and immunohistochemistry, we find IL-10, IL-3, and IL-4 in murine spongiotrophoblast mainly, with traces in the decidua (Cayol, DEA Paris 7, Sept 1997, and in preparation; See also photos 1,2, and 3), in agreement with what is reported in humans.[128,129]

### STRESS-INDUCED ABORTION

In a series of models, we have studied stress-induced abortion. Stress (be it contention, ultrasonic stress, etc.) can induce abortion in mice, and this can be prevented by alloimmunisation.[145] This phenomenon has been studied in detail. It involves pathways which are by some aspects very similar to those discussed above but involving substance P, CD8 T cells, and alterations of mast cells. The discussion of stress-induced pathways could be by itself a review, and readers should refer to other published papers.[158–165]

This model is of importance in discussing early human pregnancy loss. Of interest is the fact that a monoclonal antibody, BA 11, prepared by R. Jalali in James Mowbray laboratory, blocks *both* classical and stress-mediated abortion.[166,167]

To finish, we would like to say we do *not* negate systemic events, we simply relativise them. As stated at the beginning of this paper, we do not negate the existence of allograft enhancement during pregnancy, nor peripheral T-cell hyporesponsiveness.[26–29,67,168–170] We merely believe they reflect local events, for the most part, including the hitherto undiscussed placental suppresser factors. These, that we have suspected since 1980,[171] have proved to be elusive,[172–174] but with a continuous effort in mice,[175–182] we have delineated the active moiety in human and murine placental supernatants, and this induces T-cell anergy[181,182] in a very similar manner to staphyloccocal enterotoxin,[183] explaining, in our view, the T-cell unresponsiveness observed locally[184] whose aforementioned phenomenons (especially the report by Tafuri[27]) are in our opinion a mere reflection. In that respect, since the anergy is *transient* and reversible, it is interesting to note that optimal secretion of the factor in mice is seen by cells of the invasive ECP,[172] and that there is a local deficiency in suppresser materials in the CBA x DBA/2 window of abortion.[173]

### CONCLUSIONS

There are *several* causes of early pregnancy loss, which we are only beginning to uncover. Several, as in the murine system, have different etiologies, *but* use a *final* "rejection-like" common pathway, where TNF and gamma interferon are important. But several others are likely to be due to NK failure

TABLE 7. Effect of anti-granulocyte antibody on abortion rate

| Group | Day 6.5 treatment | Day 7.5 treatment | Day 13.5 assay | N resorptions/total | % abortion |
|---|---|---|---|---|---|
| 1 | Control rat IgG[a] | PBS | 8 | 17/57 | 30% |
| 2 | Rat anti-granulocyte[b] | PBS | 6 | 7/45 | 16%[c] |
| 3 | Control rat IgG[a] | TNF-α + γ-IFN | 9 | 48/56 | 86%[d] |
| 4 | Rat anti-granulocyte | TNF-α + γ-IFN | 7 | 8/57 | 14%[e] |
| 5 | rIL-10 | rIL-10 | 5 | 2/42 | 5%[f] |

[a]Monoclonal isotype control IgG 100 mg intraperitoneally as in Materials and Methods.
[b]Low endotoxin rat monoclonal IgG 100 mg intraperitoneally as in Materials and Methods.
[c]Reduction in abortion rate compared to group 1, $P = 0.072$ by Fisher's Exact test.
[d]TNF-α + γ-IFN given as in Tables 1 and 2. Significant increase in abortion rate compared to group 1, $P < 0.001$ by $\chi^2$ and Fisher's Exact test.
[e]Significant reduction in abortion rate compared to group 3, $P < 0.001$ by $\chi^2$ and Fisher's Exact test. Pooled result from groups 2 and 4, 15/102 = 15% abortion rate, significantly less than 30% rate in group 1, $P < 0.05$ by $\chi^2$.
[f]Significant reduction in abortion rate compared to group 1, $P < 0.0012$ by Fisher's Exact test. No significant reduction compared to group 2 ($P = 0.096$) or pooled groups 2 and 4 ($P = 0.075$).
Clark DA, Chaouat G, Arck P, Mittruecker HW, Levy GA: Cytokine-dependent abortion in CBA X DBA/2 mice is mediated by the procoagulant flg/2 prothrombinase. In press.
Stoppacciaro A, Melani C, Parenza M, et al.: Regression of an established tumor genetically modified to release granulocyte colony-stimulating factor requires granulocyte-T cell cooperation and T cell-produced interferon γ. J Exp Med 178:151, 1997.

or misrecognition, hence improper cytokine secretion and lack of growth factors.

Several others are due to defects of the local inflammatory reaction (endometriosis, with preexisting *high* levels of TNF, is among these), and it is likely that, as in the LIF system, we will see discrete defects leading to definition of molecular abnormalities, with defects in adhesion molecules being involved.

Thus, early pregnancy loss and recurrent miscarriage, though due to "immunological-like" circuitry, are likely to be split up into different syndromes. It is our opinion in that respect that it is not *at all* surprising that only one woman out of 10 or 11 benefits from purely imunologic treatment. We have to be able to define which women will benefit by rigorous immunological criteria. (Unfortunately, the leukocyte immunisation saga has lead to many charlatan, unconscious, or preposterous theorisations, sometimes with disastrous consequences; women are *not* guinea pigs nor mice! We have always wondered what was the real basis for the MHC linked, disequilibrium antigen, as well as how a system like the CBA xDBA/2–which was described as minor loci dependent, MHC restricted and in which the good father (BALB/c) and the bad father (DBA/2) were *both* H-2d–could have been taken as an example of the proof for the need for absence of HLA homology! This is just an example, but we could—and, in fact, we are due to—write horrid things about anti-paternal antibodies, to illustrate what was said at this paper's onset.)

This has to be said, because otherwise what we learn from the discoveries of complex cytokine networks at present is that by unfounded treatments we are at risk of altering other useful pathways and induce the lack of remission of transient infertility.

The "stress" saga tells us about the importance of neuroimmunoendocrine pathways, which also should not be a surprise.

To the impatience of clinicians, we recall what H. Metzger said when he was cloning the IgE receptor: "Haste is waste." We believe we have made significant progress in the past five years in understanding, *with the help of animal models*, in unraveling pathways which are operational indeed.

*But, we now have to take into account that added level of complexity.* We know it sounds difficult for clinicians, because we have a jargon, but we cannot escape the truth. *Yes, a cytokine network is operating in early pregnancy*, and, in certain cases, we might be found guilty if we were treating patients as if ignoring its existence.

### REFERENCES

1. Medawar PB: Some immunological and endocrinological problems raised by the evolution of viviparity in vertebrates. Symp Soc Exp Biol 7:320–338, 1953.
2. Brent L: A History of Transplantation Immunology. London: Academic Press, 1996.
3. Billingham RE: Immunobiological aspects of the foeto maternal relationship. In: Lachman P, Peters D (eds): Clinical Aspects of Immunology. 4th ed. Oxford: Blackwell Scientific,1981.
4. Lanman JT, Dinenstein J, Fikring S: Homograft immunity in pregnancy. Lack of harm to foetus from sensitisation of the mother. Ann NY Acad Sci 99:706–718,1962.

5. Mitchison NA: The effect on the offspring of maternal immunisation in mice. J Genet 5:406–411, 1953.
6. Monnot P, Chaouat G: Systemic active suppression is not necessary for successful allopregnancy. Am J Reprod Immunol 6:5–8, 1984.
7. Chaouat G, Voisin GA, Escalier D, Robert P: Facilitation reaction (enhancing antibodies and suppressor cells) and rejection reaction (sensitised cells) from the mother to the paternal antigens of the conceptus. Clin Exp Immunol 35:13–24, 1979.
8. Rodger JC: Lack of requirement for a maternal humoral immune response to establish or maintain a successful allogeneic pregnancy. Transplantation 40:372–375, 1985.
9. Mattson R, Mattson A, Sulila P:. Allogeneic pregnancy in B cell depleted CBA/Ca mice. Effects on fetal survival and maternal lymphoid organs. Dev Comp Immunol 9:709–717, 1985.
10. Mattson R, Holmdal R: Maintained allopregnancy in rats depleted of T cytotoxic/suppressor cells by OX8 monoclonal antibody treatment. J Reprod Immunol 1987.
11. Chaouat G, Kiger N, Wegmann TG: Vaccination against spontaneous abortion in mice. J Reprod Immunol 389–392, 1983.
12. Chaouat G, Kolb JP, Kiger N, Stanislawski M, Wegmann TG: Immunological concomitants of vaccination against abortion in mice. J Immunol 134:1594, 1985.
13. Wegmann TG: Fetal protection against abortion:is it immunosuppression or immunostimulation? Ann Immunol Inst Pasteur 135D:309, 1984.
14. Athanassakis I, Bleackley RC, Paetkau V, Guilbert L, Barrp J, Wegmann TG: The immunostimulatory effects of T cells and T cell lymphokines on murine foetally derived placental cells. J Immunol 138:37–44, 1987.
15. Wegmann TG, Athanassakis I, Guilbert L, et al.: Maternal T cell reactivity as a positive determinant of placental growth and fetal survival. In: Belissarion R, Mijezewski G (eds.): Transplantation Disorders. Prenatal Detection, Treatment and Management. New York: Alan Liss, pp. 69–76, 1991.
16. Chaouat G, Menu E,Athanassakis I, Wegmann TG: Maternal T cells regulate placental size and fetal survival. Regional Immunol 1:143, 1988.
17. Chaouat G, Menu E, Dy M, Minkowski M, Clark DA, Wegmann TG: Control of fetal survival in CBA × DBA/2 mice by lymphokine therapy. J Fertil Steril 89:447–458, 1990.
18. Clark DA,Chaput A,Tutton B: Active suppression of host versus graft reaction in pregnant mice.VII. Spontaneous abortion of CBA × DBA/2 foetuses in the uterus of CBA/J mice correlates with deficient non-T suppressor cell activity. J Immunol 136:1668–1771, 1986.
19. Clark DA, Flanders KC, Banwatt D, et al.: Murine pregnancy decidua produces a unique immunosuppressive molecule related to transforming growth factor b-2. J Immunol 144–12:3008–3004, 1990.
20. Romero R, Mazor M,Tartakowsky B: Systemic administration of Interleukin 1 induces preterm parturition in mice. Am J Obstet Gynecol 165:969–971, 1991.
21. Chwalicz K, Bukowski R, Benson M, Sdholz P, Hengele Hartung C: Labour induction, cervical ripening, and cytokines: Experimental studies in guinea pigs. In: Early embryo development, uterus preparation and role of cytokines in implantation and labour. Lyon: Editions Fondation Marcel Mérieux, pp. 165–187, 1995.
22. Romero R, Ceska M, Avila CA, Mazor M, Behnke E, Lindley J: Neutrophil attractant activating peptide/ interleukin 1/interleukin 8 and preterm parturition. Am J Obstet Gynecol 160:117–123, 1991.
23. Kelly RW, Leask R, Calder AA: Chorio decidual production of Interleukin 8 and mechanism of parturition. Lancet 339:776–777, 1993.
24. Rongiéres-Bertrand C, Menu E, Lelaidier C, Delage G, Chaouat G, Frydman R: TNF et déclenchement du travail. Contracept Fertil Steril 12:51–57, 1996.
25. Bukowski R, Scholz P, Hasan S.H, Chwalisz C: Induction of preterm parturition with the interleukin 1 beta Abst P 420, Tumor necrosis factor alpha, and with LPS guinea pigs. Soc Gyn Investigat, Toronto, Ontario, 1993.
26. Voisin G, Chaouat GA: Demonstration, nature and properties of antibodies eluted from the placenta and directed against paternal antigens. J Reprod Fertil (suppl 21):89, 1974.
27. Tafuri A, Alferink J, Molër P, Hämmerling G, Arnold B: T cell awareness of paternal alloantigens during pregnancy. Science 270:630–633, 1995.
28. Kaliss N, Dagg NK: Immune responses engendered in mice by multiparity. Transpl 2: 415–420, 1964.
29. Smith RN, Powell AE: The transfer of pregnancy induced hyporesponsiveness to male skin grafts with thymus dependent spleen cells. J Exp Med 156:899–911, 1977.
30. Tranchot Diallo J, Gras G, Parnet Mathieru F, et al.: Modulation of cytokine expression in pregnant women. Am J Reprod Immunol 37:.215–227, 1997.
31. Loke YW, King A: Human implantation. Cell Biology and Immunology. Cambridge: Cambridge University Press, 1995.
32. Karre KA: How to kill a foreign submarine. Immunol Rev 158:3–18, 1997.
33. Ellis SA: HLA-G: at the interface. Am J Reprod Immunol 23:84–86, 1990.
34. Kovatts S, Main EK, Libbrach C, Stublebline M, Fischer SJ, De Mars R: A class I antigen, HLA-G, expressed in human trophoblasts. Science 248:220–223, 1990.
35. Van Der Ven K, Ober C: HLA -G polymorphism in African Americans. J Immunol 153:5628, 1993.
36. Kovatts S, Librach C, Fisch P, et al.: The role of non classical MHC class I on human trophoblast. In: Chaouat G, Mowbray J (eds): Biologie Cellulaire et Moléculaire de la Relation Materno Fetale. Paris: Editions INSERM John Libbey, pp. 41–51, 13–21, 1991.

37. King A, Loke YW, Chaouat G: NK cells and reproduction. Immunol Today. 18:64–66, 1997.
38. King A, Hiby SE, Verma S, Buroows L, Gardner L, Loke YW: Uterine NK cells and expression of trophoblast HLA Class I molecules. Am J Reprod Immunol 37:459–463, 1997.
39. Soubiran P, Zapitelli JP, Schaffer L: IL-2 like material is present in human placenta and amnion. J Reprod Immunol 12:225–234, 1987.
40. Stern PL, Bereford N, Friedman CI, Stevens VC, Risk JM, Johnson PM: Class I like molecules are expressed by baboon placental syncytiotrophoblast. J Immunol 138:1088–1091, 1987.
41. Boyson JE, Iwanaga KK, Golos TG, Watkins DJ: Identification of rhesus monkey HLA-G ortholog. Mamu-G is a pseudo gene. J Immunol 157:5428–5437, 1996.
42. Faulk WP, Temple A: Distribution of beta 2 macroglobulin and HLA in chorionic villi of human placenta. Nature (London) 262:799–802, 1976.
43. Faulk WP, Mcintyre JA: Immunolgical studies oftrophoblast markers, subsets and functions. Immunol Rev 75:139–175, 1983.
44. Allen WR, Kydd JH, Antczack DF: Successful application of immunotherapy to a model of pregnancy failure in equids. In: Clark DA, Croy BA (eds.): Reproductive Immunology. Elsevier pp. 253–261, 1986.
45. Singh B, Raghupathy R, Anderson DJ, Wegman TG:. The murine placenta as an immunological barrier between the mother and the fetus. In: Wegmann TG, Gill TJ (eds.): Immunology of Reproduction, 51st Banff conference. New York: Oxford University Press. pp. , 1983.
46. Wegmann TG, Mossman TR, Carlson G, Olingk O, Singh B: The ability of the murine placenta to absorb monoclonal anti fetal H-2 K antibody from the maternal circulation. J Immunol 122:270–277, 1979.
47. Athanassakis Vassiliadis I, Papamettheakis J: Modulation of class II antigens on fetal placenta lads to fetal abortion. In: Chaouat G, Mowbray J (eds.): Biologie Cellulaire et Moléculaire de la Relation Materno Fetale. Paris: Editions INSERM John Libbey, pp. 69–81, 1991.
48. Mattsson R, Holmdahl R, Scheynius A, Bernadotte F, Mattsson L: Placental MHC class I antigen expression is induced in mice following in vivo treatment with recombinant interferon gamma. J Reprod Immunol 19: 115–129, 1991.
49. Clark DA: Controversies in reproductive immunology. Crit Rev Immunol 11:215, 1991.
50. Croy BA, Gambel P, Rossant J, Wegmann TG: Characterisation of murine decidual Natural Killer cells and their relevance to the success of pregnancy. Cell Immunol l93: 315–326, 1985.
51. Croy BA, Rossant J: Mouse embryonic cells become susceptible to CTL mediated lysis after midgestation. Cell Immunol 104:355–365, 1987.
52. Zuckerman FA, Head JR: Murine trophoblast resist cell mediated lysis. I. Resistance to allospecific cytotoxic T lymphocytes. J Immunol 139:2856–2865, 1987.
53. Zuckerman FA, Head JR: Murine trophoblast resist cell mediated lysis. II. Resistance to Natural cell mediated cytotoxicity. Cell Immunol 116:274–286, 1988.
54. Drake BL, Head JR: Murine trophoblast cells are susceptible to Lymphokine Activated killer (LAK) cell lysis.Am J Reprod Immunol 16:114, 1988.
55. Noun A, Acker G, Chaouat G, Antoine JC, Garabedian M. Macrophages and T lymphocyte bearing antigens bearing cells in the uterus before and during ovum implantation in the rat. Clin Exp Immunol 78:434–438, 1989.
56. Kachkache M, Acker GM, Chaouat G, Noun A, Garabedian : Hormonal and local factors control the immuno histochemical distribution of immunocytes in the rat uterus before conceptus implantation: Effects of ovariectomy, fallopian tube section and RU 486 injection. Biol Reprod 45:860–865, 1991.
57. Mac Master MT, Newton RC, Sudhanski K. Dey,and Andrews G.K. Activation and distribution of inflammatory cells in the mouse uterus during the preimplantation period. J Immunol148. 1699–1705.1992
58. Sanford TR, De M, Wood G: Expression of colony stimulating factors and inflammatory cytokines in the uterus if CD1 mice during days 1 to days 3 of pregnancy. J Reprod Fertil 94:213–220, 1992.
59. Arceci B, Shanahan F, Stanley ER, Pollard JW: The temporal expression and localisation of colony stimulating factor (CSF-1) and its receptor in the female reproductive tract are consistent with CSF-1 regulated placental development. Proc Nat Acad Sci 86:8811–8818, 1989.
60. Pollard JW, Hunt JW, Wiktor Jedrzejczack W, Stanley ER: A pregnancy defect in the osteopetrotic (op/op) mouse demonstrates the requirement for CSF-1 in female fertility. Dev Biol 148:273–283, 1991.
61. Cohen PE, Chisholm O, Arceci RJ, Stanley ER, Pollard JW: Abscence of CSF1 in osteopetrotic (csfmop/csfmop) mice results in male fertility defects. Biol Reprod 55:310– 317, 1996.
62. Pollard JW, Pampfer S, and Arceci RJ: Class III tyrosine kinase receptors at materno fetal interface. In: Chaouat G, Mowbray J (eds): Biologie Cellulaire et Moléculaire de la Relation Materno Fetale. Paris: Editions INSERM John Libbey, pp. 81–91, 1991.
63. Tartakowsky B, Goldstein O, Ben Yair B: In vivo modulation of pre embryonic development by cytokines. In: Chaouat G, Mowbray J (eds): Biologie Cellulaire et Moléculaire de la Relation Materno Fetale. Paris: Editions INSERM John Libbey, pp. 239–245, 1991.
64. Robertson SA, Seamark RF: Uterine granulocyte macrophage colony stimulating factor in early pregnancy: cellular origin and potential regulators. In: Chaouat G, Mowbray J (eds): Biologie Cellulaire et Moléculaire de la Relation Materno Fetale. Paris: Editions INSERM John Libbey, pp. 113–121, 1991.
65. Robertson SA, Seamark RF, Guilbert LJ, Wegmann TG: The rôle of cytokines in gestation. Crit Rev Immunol 14:239–292, 1994.

66. Athanassakis I, Chaouat G, Wegmann TG: The effects of anti CD4 and anti CD8 antibody treatment on placental growth and function in allogenic and syngenic pregnancy. Cell Immunol 129: 1990.
67. Robertson SA, Mau VJ, Hudson SN, Tremellen KP: Cytokine leukocyte networks and the establishment of pregnancy. Am J Reprod Immunol 37:438–443, 1997.
68. Simon C, Frances A, Piquette GN, et al.: IL-1 receptor antagonist prevents successful implantation in mice. Endocrinol 134:521–528, 1994.
69. Stewart C, Kaspar P, Brunet LJ, et a.: Blastocyst implantation depends on maternal expression of Leukemia Inhibitory Factor. Nature 359:76–79, 1992.
70. Simon C, Piquette GN, Frances A, Polan ML: Localisation of Interleukin 1 type 12 receptor and interleukin 1beta in human endometrium throughout the menstrual cycle. J Clin Endocrinol Metab 77:594–595, 1993.
71. Kauma S, Matt D, Strom S, Eirman D, Tuner T: Interleukin 1 beta (IL-1 beta), human leucocyte antigen HLA-DR alpha, and transforming growth factor beta (TGF beta) expression in endometrium, placenta and placental membranes. Am J Obstet Gynecol 163:1430–1437, 1990.
72. Simon C, Frances A,Yon Lee B, et al.: Immuno histo chemical localisation, identification and regulation of the IL 1 receptor antagonist in the human endometrium. Molec Hum Reprod 1:2472–2477, 1995.
73. Simon C, Frances A, Piquette GN, Zurawsky G, Deng W, Polan ML: Interleukin 1 system in the materno trophoblast unit in human implantation. Immuno histo chemical evidence for autocrine/paracrine function. J Clin Endocrinol Metab 878:847–854, 1994.
74. Frank GR, Brar AK, Jirihara H, Cedars MI, Handweger S: Interleukin 1 beta and endometrium: an inhibitor of stromal cell differentiation and possible auto regulator of decidualisation in human. Biol Reprod 52:184–191, 1995.
75. Steelee GL, Currie WD, Leung E, Yuen BH, Leung PCK: Rapid stimulation of Human chorionic gonadotrophin secretion by interleukin 1 beta from perfused first trimester trophoblast. J Clin. Endocrinol Metab 75:783–788, 1992.
76. Baranao RI, Piazza A, Rumi L, Polak de Fried E: Predictive value of interleukin 1 beta in supernatants of human embryo culture. Fertil Stertil 58:Abstr 0-007, 1992.
77. Sheth KV, Roca GL, Al Seidary ST, Parhar RS, Hamilton CJCM, Al-Abdul Jabbar F: Prediction of successful implantation by measuring interleukin 1 and immuno suppressive factor(s) in pre implantation embryo culture fluids. Fertil Steril 55:952–957, 1991.
78. Seifer DB, Romero R, Berlinsky D, Haning RV Jr.: Absence of cytokine production by pre implantation human embryo. Adv Res Int 30:105–107, 1993.
79. Simon C, Mercader A, Gimeno MJ, Pellicer A: The interleukin 1 system and human implantation. Am J Reprod Immunol 37:64–72, 1997.
80. Arici A, Engin O, Attar E, Olive D. Modulation of Leukemia Inhibitory Factor gene expression and protein biosynthesis in human endometrium. J Clin Endocrinol Metab 80:1908–1915, 1995.
81. Kojima K, Kanzaki H, Iwai M, et al.: Leukemia Inhibitory factor (LIF) gene expression in human endometrium. In: Nagata Y (ed): Proceedings of the 7th Annual Meeting of Japanese Society for Immunology of Reproduction. Kagoshima: pp. 245–248, 1993.
82. Kojima K, Kanzaki H, Iwai M, et al.: Expression of Leukemia Inhibitory Factor in human endometrium and placenta. Biol Reprod 50:882–887, 1994.
83. Stewart CL: A cytokine regulating embryo implantation. Second Conference on the Endometrium. Bologna. Abstract 93. 1993
84. Arici A, Engin O, Attar E, Olive D: Modulation of Leukemia Inhibitory Factor gene expression and protein biosynthesis in human endometrium. J Clin Endocrinol Metab 80:1908–1915, 199
85. Charnock-Jones DS, Sharkey A, Fenwick P, Smith SK: Leukaemia Inhibitory Factor mRNA concentration peaks in human endometrium at the time of implantation and the blastocyst contains mRNA for the receptor at this time. J Reprod Fertil 101:421–426, 1994.
86. Bhatt H, Brunet LJ, Stewart CL: Uterine expression of leukemia inhibitory factor coincides with the onset of the blastocyst implantation. Proc Natl Acad Sci 88: 11408–11412, 1991.
87. Delage G, Moreau J-F, Taupin J-L, et al.: In vitro endometrial secretion of human interleukin for DA cells/leukaemia inhibitory factor by explant cultures from fertile and infertile women. Hum Reprod 10: 2483–2488, 1995.
88. Delage G, Moreau J-F, Taupin J-L, et al.: Modulation in vitro de la production de la cytokine HILDA/LIF sécrétée par des explants d'endomètre humain: résultats préliminaires. Contracept Fertil Sex 23:622–625, 1995.
89. Laird SM, Tuckerman EM, Dalton CF, Dunphy BC, Li TC, Zhang X: The production of leukemia inhibitory factor by human endometrium: presence in uterine flushings and production by cells in culture. Hum Reprod 12:569–574, 1997.
90. Taupin J-L, Acres B, Dott K, et al.: Immunogenicity of HILDA/LIF either in a soluble or in a membrane anchored form expressed in vivo by recombinant vaccinia viruses. Scand J Immunol 38:293–301, 1993.
91. Burrows TD, King A, Loke YW: Expression of integrins by human trophoblasts and differential adhesion to laminin and fibronectin. Hum Reprod 8:475–484, 1993.
92. Burrows TD: Cell/cell and cell/matrix interactions in human placental implantation. Phd Thesis, University of Cambridge, UK, 1994.
93. Burrows TD, King A, Loke YW: Human trophoblast adhesion to matrix proteins. Inhibition ands signal transduction. Hum Reprod 10:2489–2500, 1995.
94. Zhou Y, Damsky CH, Chio K, Roberts JM, Fisher SJ: Preeclampsia is associated with abnormal expression of adhesion molecules by invasive cytotrophoblasts. J Clin Invest 91:950–960, 1993.
95. Yang Y,Yelavarthi KK, Chen HL, Pace JL, Terranova PL, Hunt JS. Molecular, biochemical and functional characteristics of tumor necrosis factor alpha produced by human placental cytotrophoblast cells. J Immunol 150:5613–5624, 1993.
96. Van de Stolpe A, Caldehoven E, Stade BG: 12-O-tetradecanoyl phorbol 13 acetate and tumor necrosis factor alpha mediated induction of intercellular adhesion molecule 1 is inhibited by dexamethasone. J Biol Chem 269:184–189, 1994.
97. Daiter E, Pampfer S, Yeung YG, Barad D, Stanley ER, Pollard JW: Expression of colony-stimulating factor-1 in the human uterus and placenta. J Clin Endocrinol. Metab 74:850–858, 1992.
98. Wu M-C, Yunis AA: Common pattern of two distinct types of colony-stimulating factor in human tissues and cultured cells. J Clin Invest 65:772–775, 1980.
99. Kanzaki H, Yui J, Iwai M: The expression and localization of mRNA for colony-stimulating factor (CSF)-1 in the human term placenta. Hum Reprod 7:563–567, 1992.
100. Pampfer S, Tabibzadeh S, Chuan FC, Pollard JW: Expression of colony-stimulating factor-1 (CSF-1) messenger RNA in human endometrial glands during the menstrual cycle; molecular cloning of a novel transcript that predicts a cell surface form of CSF-1. Mol Endocrinol 5:1931–1938, 1991.
101. Kariya M, Kanzaki H, Hanamura T: Progesterone-dependent secretion of macrophage colony-stimulating factor by human endometrial stromal cells of non-pregnant uterus in culture. J Clin Endocrinol Metab 79:86–90, 1994.
102. Shorter SC, Vince GS, Starkey PM: The identification of mRNA for the IL-3 related cytokines in placental and decidual tissues. In: Cedard L, Chaouat G, Challier JC, Neismann C (eds): Proceedings of the Third Meeting of the European Placenta Group. Paris: INSERM. John Libbey Eurotexte, p. 145, 1989.
103. Martal J, Assal NE, Assal Meliani A: Immuno endocrine properties of tau interferons in the maternal recognition of pregnancy. In: Glasser SR (eds): Endocrinology of Embryo Endometrium Interactions. Ciba Foundation Symposium. International Congress of Endocrinology, Bordeaux. Ciba Foundation Editors. New York: Plenum Press, pp. 195–216, 1993.
104. Bazer FW, Spencer TE, Ott TL: Interferons tau: a novel pregnancy recognition signal. Am J Reprod Immunol 412–421, 1997.
105. Fillion C, Chaouat G, Reinaud P, Charpigny JC, Martal J: Immunoregulatory effects of trophoblastin (oTP): all 5 isoforms are immuno suppressive of PHA driven lymphocyte proliferation. J Reprod Immunol 19:237–249, 1991.
106. Assal-Meliani A, Charpigny G, Reinaud P, Martal J, Chaouat G: Recombinant ovine trophoblastin (r.otp) inhibits ovine, murine and human lymphocyte proliferation. J Reprod Immunol 25:148–165, 1993.
107. Roberts RM, Cross JC, Leaman DW: Interferons as hormones of pregnancy. Endocr Rev 13:432–452, 1992.
108. Chaouat G, Assal-Meliani A, Martal J: IL-10 prevents naturally occuring foetal loss in the CBA x DBA/2 mating combination, and local defect in IL-10 production in this abortion-prone combination is corrected by in vivo injection of IFN-t. J Immunol 154:4261–4268, 1995.
109. Whaley AE, Reddy Meka CS, Harbison LA, Hunt JS, Imakawa K: Identification and cellular localisation of unique interferon mRNA from human placenta. J Biol Chem 269:10864–10868, 1994.
110. DW, Hunter C, Mitchell MD, Warner MW, Gately M: Elevation of serum IL 12 levels in women with severe pre eclampsia and HELLP syndrome. J Dudley Reprod Immunol 31: 1–2, 97–108, 1996.
111. Sunder Plassman G, Derfler K, Wagner L: Increased serum activity of Interleukin 2 in patients with pre-eclampsia. J Autoimmun 2:203–205, 1989.
112. Boehm KD, Kelley MK, Ilan J, Ilan J The interleukin 2 gene is expressed in the syncytiotrophoblast of the human placenta. Proc Nat Acad Sci 86:656–660, 1996.
113. Tezabwala BU, Johnstone AP: Effects of administration of interleukin 2 in pregnancy. J Reprod Immunol (suppl)1:147, 1986.
114. Lala PK, Parhar RS, Kearns M, Johson S, Scodras JM: Immunological aspects of the decidual response. J Reprod Immunol (suppl) 1:20, 1986.
115. Mattsson R, Holmdahl R, Scheynius A, Bernadotte F, Mattsson L: Placental MHC class I antigen expression is induced in mice following in vivo treatment with recombinant interferon gamma. J Reprod Immunol 19: 115–129, 1991.
116. Hunt JS, Hua Lin C, Miler L. Tumor necrosis factors: pivotal components of pregnancy? Biol Reprod 54:554–563, 1996.
117. Parand M, Chedid L: Protective effects of chlorpromazine against endotoxin induced abortion. Proc Soc Exp Biol Med 116:906–915, 1964.
118. Beaman KD, Hoversland RC: Induction of "spontaneous" abortion by blocking antigen specific suppression. J Reprod Fertil 82:135–139, 1988.
119. Hoversland RC, Beaman KD: Embryo implantation associated with increase in T cell suppressor factor in the uterus and spleen of mice. J Reprod Fertil 88:135–139, 1990.
120. Rubesa G, Beaman KD, Beer AE, Haller H, Rukavina D: Expression of membrane associated protein TJ 6 on decidual lymphocytes in the first trimester of pregnancy. J Reprod Immunol 30:12–27, 1996.
121. Beaman K, Angkachatchai V, Gilman Sachs A: TJ 6, the pregnancy associated cytokine. Am J Reprod Immunol 35:338–342, 1996.
122. Szekeres Bartho J: Immunosuppression by Progesterone in Pregnancy. Boca Raton: CRC Press, 1992.
123. Szekeres Bartho J, Wegmann TG: A progesterone dependent immunomodulatory protein alters the Th1/Th2 balance. J Reprod Immunol 31:81–97, 1996.
124. Szekeres Bartho J, Faust P, Varga P, Szereday L, Kelemen K: The immunolgical pregnancy protective effect of progesterone is manifested via control of cytokine production. Am J Reprod Immunol 35:348–352, 1996.

125. Check JH, Arwitz M, Gross J, Szekeres Bartho J, Wu CH: Evidence that expression of progesterone induced blocking factor by maternal T lymphocytes is positively correlated with conception. Am J Reprod Immunol 38:6–9, 1997.
126. Leah RG, Vince G, Flanders KC, et al.: Uterine TGF beta 2 in human normal and pathologic pregnancies. In: Early Embryo Development, Uterus Preparation and Role of Cytokines in Implantation and Labour. Lyon: Editions Fondation Marcel Mérieux, pp., 1995.
127. Wegmann TG, Lin H, Guilbert L, Mossman TH: Bidirectional cytokines interactions in the materno fetal relationship: successful allopregnancy is a Th2 phenomenon. Immunol Today 14:353–355, 1993.
128. Bennet WA, Lagoo-Dennayan S, Whitworth NS, Brackin MN, Hale E, Cowan BD: Expression and production of interleukin 10 by human trophoblast: a model for cytokine regulation of pregnancy immunotolerance. In: The Third World Conference on Early Pregnancy. New York: Parthenon Publishing, p. 75, 1996.
129. Roth I, Corry D, Locksley R, Abrams J, Litton M, Fisher S: Human placental cytotrophoblasts produce the immunosuppressive cytokine interleukin 10. J Exp Med 184: 539–548, 1996.
130. Mincheva Nillson L, Baranov V, Hammarstrom ML, Hammarstrom S: Phenotypic and functional analysis of human decidua associated lymphoid cells from normal human pregnancy. In: Early Embryo Development, Uterus Preparation and Role of Cytokines in Implantation and Labour. Lyon: Editions Fondation Marcel Mérieux, pp. 113–141, 1995.
131. Delassus S, Countinho GS, Saucier S, Darche S, Kourilky P: Differential cytokine expression in maternal blood and placenta during murine gestation. J Immunol 152:2411–2420, 1994.
132. Lin H, Mossman TR, Guilbert L, Tuntipopipat S, Wegmann TG: Synthesis of T Helper-2 cytokines at the maternal fetal interface. J Immunol 151: 4562, 1993.
133. Krishnan L, Guilbert LJ, Wegmann TG, Belosevic M, and Mosmann TR: T helper 1 response against Leishmania major in pregnant C57Bl/6 mice increases implantation failure and fetal resorptions. Correlation with increased IFN-g and TNF and reduced IL-10 production by placental units. J Immunol 156:653, 1996.
134. Krishnan L, Guilbert LJ, Russell AS, Wegmann TG, Mosmann TR, Belosevic M: Pregnancy impairs resistance of C57Bl/6 mice to Leishmania major infection and causes decreased antigen-specific IFN-g response and increased production of T helper 2 cytokines. J Immunol 156:644, 1996.
135. Tangri S, Raghupathy R: Expression of cytokines in placentas of mice undergoing immunologically mediated spontaneous foetal resorptions. Biol Reprod 49: 850–856, 1993.
136. Tangri S, Wegmann TG, Lin H, Raghupathy R: Maternal anti placental activity in natural, immunologically mediated fetal resorption. J Immunol 152:4903–4910, 1994.
137. Hill J: T helper 1 immunity to trophoblast: evidence for a new immunological mechanism for recurrent abortion in women Hum Reprod 10:114–120, 1995.
138. Hill JA, Polgar K, Anderson DJ: Helper 1 type immunity in women with recurrent spontaneous abortion. JAMA 273:1993–1935, 1995.
139. Guimond MJ, Luross JA, Wang B, Terhorst C, Danial S, Croy BA: 107.Abscence of natural killer cells during murine pregnancy is associated with reproductive compromise in TgE26 mice. Biol Reprod 56:169–17
140. Chaouat G, Tranchot Diallo J, Volumenie JL, et al.: Immune suppression and Thl /Th2 balance in pregnancy revisited. A (very) personal tribute to Tom Wegman. Am J Reprod Immunol 37:427–435,.
141. Chaouat G, Clark DA, Wegmann TG: Genetics aspects of the CBA/J x DBA/2 J and B10 x B10.A models of murine spontaneous abortions and prevention by leukocyte immunisation. In: RCOG, Allen WR, Clark DA, Gill TJ III, Mowbray JF, Robertson WR (eds.): 18 th RCOG Study Group. Early Pregnancy Loss. Mechanisms and Treatment. RCOG Press, pp.89–105, 1988.
142. Kinsky R, Delage G, Rosin N, Thang MN, Hoffmann M, Chaouat G. A murine model of NK mediated foetal resorbtion. Am J Reprod Immunol 23:73–78, 1990.
143. Chaouat G, Menu E, David F, Djian V, Kinsky R: Reproductive immunology 1989–1992: some important recent advances about feto maternal relationship. In: Gergely J (ed.) Progress in Immunology 8 (8th International Congress Immunology) Springer Verlag, p. 825, 1993.
144. Kinsky R, Kapovic M, Menu E: The rôle of defined major histocompatibility antigens in preventing foetal death. In: The Immunology of Pregnancy. Boca Raton: CRC Press, pp. 47–61, 1993.
145. Chaouat G: Synergy of lipopolysaccharide and inflammatory cytokines in murine pregnancy: alloimmunization prevents abortion but does not affect the induction of preterm delivery. Cell Immunol 157:328, 1994.
146. Gendron RL, Nestel FP, Lapp WS, Baines MG: Lipopolysaccharide induced fetal resorption in mice is associated with the intrauterine production of tumor necrosis factor-α. J Reprod Fertil 90:447, 1990.
147. Duclos AJ, Haddad EK, Baines MG: Presence of activated macrophages in a murine model of early embryo loss. Am J Reprod Immunol 33:354, 1995.
148. Haddad EKA, Duclos J, Lapp WS, Baines MG: Early embryo loss is associated with the prior expression of macrophage activation markers in the decidua. J Immunol 158:4886, 1997.
149. Haddad EK, Duclos AJ, Baines MG: Early embryo loss is associated with local production of nitrc oxyde by activated macrophages. J Exp Med 182:1143–1152, 1995.

150. Baines MG, Duclos AG, Antecka E, Haddad EK: Decidual infiltration and activation of macrophages lead to early embryo loss. Am J Reprod Immunnol 37:471–478, 1997.
151. De Fougerolles R, Baines M: Modulation of Natural Killer activity influences resorption rates in CBA × DBA/2 matings. J Reprod Immunol 11:147, 1988.
152. Gendron R, Baines M: Infiltrating decidual Natural Killer cells are associated with spontaneous abortion in mice. Cell Immunol, 113:261, 1988.
153. Chaouat G, Menu E, Wegmann TJ: Role des lymphokines de la famille du CSF, et du TNF, de l'interféron gamma, et de l'IL-2 sur la survie fetale et la croissance placentaire étudiées in vivo dans 2 modéles d'avortements immunitaires spontanés murins. Biologie cellulaire et moléculaire de la relation materno fetale. Paris: Editions INSERM John Libbey, pp. 91–101, 1991.
154. Chaouat G, Menu E, Wegmann TJ: Role des lymphokines de la famille du CSF, et du TNF, de l'interféron gamma, et de l'IL-2 sur la survie fetale et la croissance placentaire étudiées in vivo dans 2 modéles d'avortements immunitaires spontanés murins. Biologie cellulaire et moléculaire de la relation materno fetale. Editions INSERM John Libbey.Pages 91–101, 1991.
155. Clark DA, Chaouat G, Arck P, Mittruecker HW, Levy GA: Cytokine-dependent abortion in CBA X DBA/2 mice is mediated by the procoagulant fgl/2 prothrombinase. In press. Stoppacciaro A, Melani C, Parenza M, et al.: Regression of an established tumor genetically modified to release granulocyte colony-stimulating factor requires granulocyte-T cell cooperation and T cell-produced interferon g. J Exp Med 178:151, 1997.
156. Clark DA, Merali F, Hoskin D, et al.: Murine pregnancy decidua-associated suppressor cells in abortion prone DBA/2-mated CBA/J mice that release bioactive transforming growth factor b2-related immunosuppressive molecules express a bone-marrow-derived natural suppressor cell marker and gd T cell receptor. Biol Reprod 56:1351, 1997.
157. Arck P, Ferrick DA, Steele Norwood D, Croitaru K, Clark DA: Murine T cell determination of pregnancy outcome. I) Effects of strain, ab T cell receptor, gd T celll receptor and gdT cell subsets. Am J Reprod Immunol 37:492–503, 1997.
158. Clark DA, Banwatt D, Chaouat G: Stress triggered abortion in mice is prevented by alloimmunisation. Am J Reprod Immunol 29:141–148, 1993.
159. Clark DA, Chaouat G, Mogil R, Wegmann TG: Prevention of spontaneous abortion in DBA/2 mated CBA/J mice by GM CSF involves CD8+ T cell dependent suppression of Natural effector cells cytotoxicity against trophoblast target cells. Cell Immunol 154:143–153, 1994.
160. Arck P, Merali FS, Stanicz AM, et al.: Stress induced murine abortion associated with substance P dependent alterations in maternal uterine decidua. Biol Reprod 53:814–820, 1995.
161. Arck P, Merali C, Chaouat G, Clark DA: Inhibition of immunoprotective CD8+ cells as a basis for stress trigerred abortion in mice. Substance P mediated abortion in mice. Cell Immunol 171:226–230, 1996.
162. Clark DA, Arck P, Jalali R, et al.: Psycho neuro cytokin endocrine pathway in immunoregulation during pregnancy. Am J Reprod Immunol 33:330–337, 1996.
163. Arck P, Merali F, Stead R. Chaouat G, Clark DA: Stress triggered abortion: inhibition of protective suppressor mechanisms and promotion of TNF alpha release via neuro transmitter. Am J Reprod Immunol 33:74–80, 1995.
164. Markert U, Azrck CP, McBey BA, et al.: Stress triggered abortion are associated with alterations of granulated cells in tne decidua. Am J Reprod Immunol 37: 94–101, 1997.
165. Arck PC, Troutt AB, Clark DA: Soluble receptors neutralizing TNF-a and IL-1 block stress-triggered murine abortion. Am J Reprod Immunol 37:262–267, 1997.
166. Mowbray JR, Jalalai RE, Chaouat G, et al.: Maternal response to trophoblast antigens. Am J Reprod Immunol 37:421–427, 1997.
167. Jalali R, Arck P, Surridge S, et al.: Immunosupressive properties of monoclonal antibodies and human polyclonal antibodies to the R80K protein of trophoblast. Am J Reprod Immunol 36:129–134, 1997.
168. Baines MG, Speers EA, Pross HGF, Millar KG: Characteristics of the maternal lymphoid response in mice to paternal strain alloantigens induced by homologous pregnancy. Immunol 31:363–369, 1976.
169. Breyere EJ, Barret MK: Prolonged survival of skin homografts in parous mice. J Natl Cancer Institute 25: 1405–1410, 1960.
170. Baines MG, Millar KG, Pross HF: Allograft enhancement during normal murine pregnancy. J Reprod Immunol 2:141–148, 1980.
171. Chaouat G, Chaffaux S, Voisin GA: Immunoactive products of placenta (I). J Reprod Immuno. 2:127–136, 1980.
172. Culouscou JM, Remacle Bonnet MM, Pommier G, Rancer J, Depieds RC: Immunosuppressive properties of human placenta: study of supernatants from short term syncytiotrophoblast cultures. Cell Immunol 9:33, 1986.
173. Hamaoka T, Majusaki N, Itoh S: Human trophoblast and tumor cell derived immunoregulatory factor. In: Isojima S, Billington WD (eds.): Reproductive Immunology, Elsevier, pp. 133–146, 1983.
174. Saji F, Koyama M, Kameda T, Negoro T, Nakamuro K, Tanizawa O: Effect of a soluble factor secreted from human trophoblast cells on in vitro lymphocyte reactions. Am J Reprod Immunol 13:121, 1987.
175. Chaouat G, Kolb JP: Immunoactive products of murine placenta. II. Afferent suppression of maternal cell mediated immunity by supernatants from short term enriched cultures of murine trophoblast enriched maternal cell populations. Ann Immunol Institut Pasteur 135C:205–218, 1984.

176. Menu E, Kaplan L, Andreu G, Denver L, Chaouat G: Immunoactive products of human placenta, I. An Immunoregulatory factor obtained from explant cultures of human placenta inhibits CTL generation and cytotoxic effector activity. Cell Immunol 119:341, 1989.
177. Menu E, Jankovic DL, Theze J, David V, Chaouat G: Immunoactive products of human placenta, III. Characterization of an inhibitor affecting lymphocyte proliferation. Regional Immunol 3:254–259, 1991.
178. Menu E, Kinsky R, Hoffman M, Chaouat G: Immunoactive products of human placenta, IV. Immunoregulatory factors obtained from cultures of human placenta inhibit local and general murine graft versus host reactions (GVHR). J Reprod Immunol 20:195–204, 1991.
179. Menu E, Djian V, Kinsky R: Immunoactive products of human placenta. Biologie cellulaire et moléculaire de la relation materno fetale. Paris: Editions INSERM John Libbey, pp. 197–203, 1991.
180. Djian V, Menu E, Thibault G, Ropert S. Chaouat G: Immunoactive products of placenta. V. Immunoregulatory properties of a low molecular weight compound obtained from human placental cultures. Am J Reprod Immunol 36:11–24, 1996.
181. De Smedt D, Menu E, Chaouat G. Immunoactive products of placenta VI. Induction of T cell anergy by a low molecular weight compound obtained from supernatants of human placental cultures. Cell Immunol 175:128–140,
182. Voluménie J-L, Mognetti B, De Smedt D, Menu E, Chaouat G. Induction of transient murine T cell anergy by a low molecular weight compound obtained from supernatants of human placental cultures is linked to defective phosphorylation of TcR ζ CD3 chain. Am J Reprod Immunol. In press.
183. Migita K, Eguchi K, Kawabe Y, Tsukada T, Ichinose Y, Nagataki: Defective TcR chain phosphorylation in SEB induced anergic T cells. J Immunol 15:5083 –5089, 1995.
184. Mincheva-Nilsson L, Hammarström S, Hammarström ML: Human decidual leukocytes from early pregnancy contain high number of gamma-delta+ cells and show selective down regulation of alloreactivity. J Immunol 149:2203, 1992
185. Baines MG, Gendron RL: Natural and experimental animal models of reproductive failure in Immunology of Pregnancy. In: Chaouat G (ed.) Boca Raton, Florida: CRC Press. 173–203, 1993.

## References

[PDF_01442] Arceci R. J., Shanahan F., Stanley E. R., Pollard J. W. (1989). Temporal expression and location of colony-stimulating factor 1 (CSF-1) and its receptor in the female reproductive tract are consistent with CSF-1-regulated placental development.. Proc Natl Acad Sci U S A.

[PDF_01877] Arck P. C., Ferrick D. A., Steele-Norwood D., Croitoru K., Clark D. A. (1997). Murine T cell determination of pregnancy outcome: I. Effects of strain, alphabeta T cell receptor, gammadelta T cell receptor, and gammadelta T cell subsets.. Am J Reprod Immunol.

[PDF_01902] Arck P. C., Merali F. S., Manuel J., Chaouat G., Clark D. A. (1995). Stress-triggered abortion: inhibition of protective suppression and promotion of tumor necrosis factor-alpha (TNF-alpha) release as a mechanism triggering resorptions in mice.. Am J Reprod Immunol.

[PDF_01891] Arck P. C., Merali F. S., Stanisz A. M., Stead R. H., Chaouat G., Manuel J., Clark D. A. (1995). Stress-induced murine abortion associated with substance P-dependent alteration in cytokines in maternal uterine decidua.. Biol Reprod.

[PDF_01895] Arck P. C., Merali F., Chaouat G., Clark D. A. (1996). Inhibition of immunoprotective CD8+ T cells as a basis for stress-triggered substance P-mediated abortion in mice.. Cell Immunol.

[PDF_01911] Arck P. C., Troutt A. B., Clark D. A. (1997). Soluble receptors neutralizing TNF-alpha and IL-1 block stress-triggered murine abortion.. Am J Reprod Immunol.

[PDF_01536] Arici A., Engin O., Attar E., Olive D. L. (1995). Modulation of leukemia inhibitory factor gene expression and protein biosynthesis in human endometrium.. J Clin Endocrinol Metab.

[PDF_01552] Arici A., Engin O., Attar E., Olive D. L. (1995). Modulation of leukemia inhibitory factor gene expression and protein biosynthesis in human endometrium.. J Clin Endocrinol Metab.

[PDF_01652] Assal-Meliani A., Charpigny G., Reinaud P., Martal J., Chaouat G. (1993). Recombinant ovine trophoblastin (roTP) inhibits ovine, murine and human lymphocyte proliferation.. J Reprod Immunol.

[PDF_01269] Athanassakis I., Bleackley R. C., Paetkau V., Guilbert L., Barr P. J., Wegmann T. G. (1987). The immunostimulatory effect of T cells and T cell lymphokines on murine fetally derived placental cells.. J Immunol.

[PDF_01922] Baines M. G., Speers E. A., Pross H., Millar K. G. (1976). Characteristics of the maternal lymphoid response of mice to paternal strain antigens induced by homologous pregnancy.. Immunology.

[PDF_01706] Beaman K., Angkachatchai V., Gilman-Sachs A. (1996). TJ6: the pregnancy-associated cytokine.. Am J Reprod Immunol.

[PDF_01561] Bhatt H., Brunet L. J., Stewart C. L. (1991). Uterine expression of leukemia inhibitory factor coincides with the onset of blastocyst implantation.. Proc Natl Acad Sci U S A.

[PDF_01675] Boehm K. D., Kelley M. F., Ilan J., Ilan J. (1989). The interleukin 2 gene is expressed in the syncytiotrophoblast of the human placenta.. Proc Natl Acad Sci U S A.

[PDF_01371] Boyson J. E., Iwanaga K. K., Golos T. G., Watkins D. I. (1996). Identification of the rhesus monkey HLA-G ortholog. Mamu-G is a pseudogene.. J Immunol.

[PDF_01820] Brown C. R., Clarke N., Aiken M., Bavister B. D. (1990). Changes in the composition of the hamster zona pellucida after fertilization in vivo but not in vitro.. J Reprod Fertil.

[PDF_01584] Burrows T. D., King A., Loke Y. W. (1993). Expression of integrins by human trophoblast and differential adhesion to laminin or fibronectin.. Hum Reprod.

[PDF_01591] Burrows T. D., King A., Smith S. K., Loke Y. W. (1995). Human trophoblast adhesion to matrix proteins: inhibition and signal transduction.. Hum Reprod.

[PDF_01658] Chaouat G., Assal Meliani A., Martal J., Raghupathy R., Elliott J. F., Elliot J., Mosmann T., Wegmann T. G. (1995). IL-10 prevents naturally occurring fetal loss in the CBA x DBA/2 mating combination, and local defect in IL-10 production in this abortion-prone combination is corrected by in vivo injection of IFN-tau.. J Immunol.

[PDF_01948] Chaouat G., Kolb J. P. (1984). Immunoactive products of murine placenta. II.--Afferent suppression of maternal cell-mediated immunity by supernatants from short-term cultures of murine trophoblast-enriched cell suspensions.. Ann Immunol (Paris).

[PDF_01263] Chaouat G., Kolb J. P., Kiger N., Stanislawski M., Wegmann T. G. (1985). Immunologic consequences of vaccination against abortion in mice.. J Immunol.

[PDF_01280] Chaouat G., Menu E., Athanassakis I., Wegmann T. G. (1988). Maternal T cells regulate placental size and fetal survival.. Reg Immunol.

[PDF_01283] Chaouat G., Menu E., Clark D. A., Dy M., Minkowski M., Wegmann T. G. (1990). Control of fetal survival in CBA x DBA/2 mice by lymphokine therapy.. J Reprod Fertil.

[PDF_01240] Chaouat G., Monnot P. (1984). Systemic active suppression is not necessary for successful allopregnancy.. Am J Reprod Immunol.

[PDF_01816] Chaouat G. (1994). Synergy of lipopolysaccharide and inflammatory cytokines in murine pregnancy: alloimmunization prevents abortion but does not affect the induction of preterm delivery.. Cell Immunol.

[PDF_01243] Chaouat G., Voisin G. A., Escalier D., Robert P. (1979). Facilitation reaction (enhancing antibodies and suppressor cells) and rejection reaction (sensitized cells) from the mother to the paternal antigens of the conceptus.. Clin Exp Immunol.

[PDF_01556] Charnock-Jones D. S., Sharkey A. M., Fenwick P., Smith S. K. (1994). Leukaemia inhibitory factor mRNA concentration peaks in human endometrium at the time of implantation and the blastocyst contains mRNA for the receptor at this time.. J Reprod Fertil.

[PDF_01720] Check J. H., Arwitz M., Gross J., Szekeres-Bartho J., Wu C. H. (1997). Evidence that the expression of progesterone-induced blocking factor by maternal T-lymphocytes is positively correlated with conception.. Am J Reprod Immunol.

[PDF_01899] Clark D. A., Arck P. C., Jalali R., Merali F. S., Manuel J., Chaouat G., Underwood J. L., Mowbray J. F. (1996). Psycho-neuro-cytokine/endocrine pathways in immunoregulation during pregnancy.. Am J Reprod Immunol.

[PDF_01882] Clark D. A., Banwatt D., Chaouat G. (1993). Stress-triggered abortion in mice prevented by alloimmunization.. Am J Reprod Immunol.

[PDF_01885] Clark D. A., Chaouat G., Mogil R., Wegmann T. G. (1994). Prevention of spontaneous abortion in DBA/2-mated CBA/J mice by GM-CSF involves CD8+ T cell-dependent suppression of natural effector cell cytotoxicity against trophoblast target cells.. Cell Immunol.

[PDF_01287] Clark D. A., Chaput A., Tutton D. (1986). Active suppression of host-vs-graft reaction in pregnant mice. VII. Spontaneous abortion of allogeneic CBA/J x DBA/2 fetuses in the uterus of CBA/J mice correlates with deficient non-T suppressor cell activity.. J Immunol.

[PDF_01405] Clark D. A. (1991). Controversies in reproductive immunology.. Crit Rev Immunol.

[PDF_01293] Clark D. A., Flanders K. C., Banwatt D., Millar-Book W., Manuel J., Stedronska-Clark J., Rowley B. (1990). Murine pregnancy decidua produces a unique immunosuppressive molecule related to transforming growth factor beta-2.. J Immunol.

[PDF_01870] Clark D. A., Merali F. S., Hoskin D. W., Steel-Norwood D., Arck P. C., Croitoru K., Murgita R. A., Hirte H. (1997). Decidua-associated suppressor cells in abortion-prone DBA/2-mated CBA/J mice that release bioactive transforming growth factor beta2-related immunosuppressive molecules express a bone marrow-derived natural suppressor cell marker and gamma delta T-cell receptor.. Biol Reprod.

[PDF_01452] Cohen P. E., Chisholm O., Arceci R. J., Stanley E. R., Pollard J. W. (1996). Absence of colony-stimulating factor-1 in osteopetrotic (csfmop/csfmop) mice results in male fertility defects.. Biol Reprod.

[PDF_01407] Croy B. A., Gambel P., Rossant J., Wegmann T. G. (1985). Characterization of murine decidual natural killer (NK) cells and their relevance to the success of pregnancy.. Cell Immunol.

[PDF_01411] Croy B. A., Rossant J. (1987). Mouse embryonic cells become susceptible to CTL lysis after midgestation.. Cell Immunol.

[PDF_01609] Daiter E., Pampfer S., Yeung Y. G., Barad D., Stanley E. R., Pollard J. W. (1992). Expression of colony-stimulating factor-1 in the human uterus and placenta.. J Clin Endocrinol Metab.

[PDF_01565] Delage G., Moreau J. F., Taupin J. L., Freitas S., Hambartsoumian E., Olivennes F., Fanchin R., Letur-Könirsch H., Frydman R., Chaouat G. (1995). In-vitro endometrial secretion of human interleukin for DA cells/leukaemia inhibitory factor by explant cultures from fertile and infertile women.. Hum Reprod.

[PDF_01570] Delage G., Moreau J. F., Taupin J. L., Hambartsoumian E., Frydman R., Tartzkovsky B., Chaouat G. (1995). Modulation in vitro de la production de la cytokine HILDA/LIF sécrétée par des explants d'endomètre humain: résultats préliminaires.. Contracept Fertil Sex.

[PDF_01752] Delassus S., Coutinho G. C., Saucier C., Darche S., Kourilsky P. (1994). Differential cytokine expression in maternal blood and placenta during murine gestation.. J Immunol.

[PDF_01975] Djian V., Menu E., Thibault G., Ropert S., Chaouat G. (1996). Immunoactive products of placenta. V. Immunoregulatory properties of a low molecular weight compound obtained from human placental cultures.. Am J Reprod Immunol.

[PDF_01824] Duclos A. J., Haddad E. K., Baines M. G. (1995). Presence of activated macrophages in a murine model of early embryo loss.. Am J Reprod Immunol.

[PDF_01344] Ellis S. (1990). HLA G: at the interface.. Am J Reprod Immunol.

[PDF_01377] Faulk W. P., McIntyre J. A. (1983). Immunological studies of human trophoblast: markers, subsets and functions.. Immunol Rev.

[PDF_01374] Faulk W. P., Temple A. (1976). Distribution of beta2 microglobulin and HLA in chorionic villi of human placentae.. Nature.

[PDF_01647] Fillion C., Chaouat G., Reinaud P., Charpigny J. C., Martal J. (1991). Immunoregulatory effects of ovine trophoblastin protein (oTP): all five isoforms suppress PHA-induced lymphocyte proliferation.. J Reprod Immunol.

[PDF_01511] Frank G. R., Brar A. K., Jikihara H., Cedars M. I., Handwerger S. (1995). Interleukin-1 beta and the endometrium: an inhibitor of stromal cell differentiation and possible autoregulator of decidualization in humans.. Biol Reprod.

[PDF_01843] Gendron R. L., Baines M. G. (1988). Infiltrating decidual natural killer cells are associated with spontaneous abortion in mice.. Cell Immunol.

[PDF_01831] Haddad E. K., Duclos A. J., Baines M. G. (1995). Early embryo loss is associated with local production of nitric oxide by decidual mononuclear cells.. J Exp Med.

[PDF_01827] Haddad E. K., Duclos A. J., Lapp W. S., Baines M. G. (1997). Early embryo loss is associated with the prior expression of macrophage activation markers in the decidua.. J Immunol.

[PDF_01782] Hill J. A. (1995). T-helper 1-type immunity to trophoblast: evidence for a new immunological mechanism for recurrent abortion in women.. Hum Reprod.

[PDF_01698] Hoversland R. C., Beaman K. D. (1990). Embryo implantation associated with increase in T-cell suppressor factor in the uterus and spleen of mice.. J Reprod Fertil.

[PDF_01689] Hunt J. S., Chen H. L., Miller L. (1996). Tumor necrosis factors: pivotal components of pregnancy?. Biol Reprod.

[PDF_01918] Jalali G. R., Arck P., Surridge S., Markert U., Chaouat G., Clark D. A., Underwood J. L., Mowbray J. F. (1996). Immunosuppressive properties of monoclonal antibodies and human polyclonal alloantibodies to the R80K protein of trophoblast.. Am J Reprod Immunol.

[PDF_01428] Kachkache M., Acker G. M., Chaouat G., Noun A., Garabédian M. (1991). Hormonal and local factors control the immunohistochemical distribution of immunocytes in the rat uterus before conceptus implantation: effects of ovariectomy, fallopian tube section, and injection.. Biol Reprod.

[PDF_01616] Kanzaki H., Yui J., Iwai M., Imai K., Kariya M., Hatayama H., Mori T., Guilbert L. J., Wegmann T. G. (1992). The expression and localization of mRNA for colony-stimulating factor (CSF)-1 in human term placenta.. Hum Reprod.

[PDF_01626] Kariya M., Kanzaki H., Hanamura T., Imai K., Narukawa S., Inoue T., Hatayama H., Mori T. (1994). Progesterone-dependent secretion of macrophage colony-stimulating factor by human endometrial stromal cells of nonpregnant uterus in culture.. J Clin Endocrinol Metab.

[PDF_01496] Kauma S., Matt D., Strom S., Eierman D., Turner T. (1990). Interleukin-1 beta, human leukocyte antigen HLA-DR alpha, and transforming growth factor-beta expression in endometrium, placenta, and placental membranes.. Am J Obstet Gynecol.

[PDF_01312] Kelly R. W., Leask R., Calder A. A. (1992). Choriodecidual production of interleukin-8 and mechanism of parturition.. Lancet.

[PDF_01360] King A., Hiby S. E., Verma S., Burrows T., Gardner L., Loke Y. W. (1997). Uterine NK cells and trophoblast HLA class I molecules.. Am J Reprod Immunol.

[PDF_01358] King A., Loke Y. W., Chaouat G. (1997). NK cells and reproduction.. Immunol Today.

[PDF_01803] Kinsky R., Delage G., Rosin N., Thang M. N., Hoffmann M., Chaouat G. (1990). A murine model of NK cell mediated resorption.. Am J Reprod Immunol.

[PDF_01546] Kojima K., Kanzaki H., Iwai M., Hatayama H., Fujimoto M., Inoue T., Horie K., Nakayama H., Fujita J., Mori T. (1994). Expression of leukemia inhibitory factor in human endometrium and placenta.. Biol Reprod.

[PDF_01346] Kovats S., Main E. K., Librach C., Stubblebine M., Fisher S. J., DeMars R. (1990). A class I antigen, HLA-G, expressed in human trophoblasts.. Science.

[PDF_01767] Krishnan L., Guilbert L. J., Russell A. S., Wegmann T. G., Mosmann T. R., Belosevic M. (1996). Pregnancy impairs resistance of C57BL/6 mice to Leishmania major infection and causes decreased antigen-specific IFN-gamma response and increased production of T helper 2 cytokines.. J Immunol.

[PDF_01760] Krishnan L., Guilbert L. J., Wegmann T. G., Belosevic M., Mosmann T. R. (1996). T helper 1 response against Leishmania major in pregnant C57BL/6 mice increases implantation failure and fetal resorptions. Correlation with increased IFN-gamma and TNF and reduced IL-10 production by placental cells.. J Immunol.

[PDF_01232] LANMAN J. T., DINERSTEIN J., FIKRIG S. (1962). Homograft immunity in pregnancy: lack of harm to the fetus from sensitization of the mother.. Ann N Y Acad Sci.

[PDF_01575] Laird S. M., Tuckerman E. M., Dalton C. F., Dunphy B. C., Li T. C., Zhang X. (1997). The production of leukaemia inhibitory factor by human endometrium: presence in uterine flushings and production by cells in culture.. Hum Reprod.

[PDF_01756] Lin H., Mosmann T. R., Guilbert L., Tuntipopipat S., Wegmann T. G. (1993). Synthesis of T helper 2-type cytokines at the maternal-fetal interface.. J Immunol.

[PDF_01907] Markert U. R., Arck P. C., McBey B. A., Manuel J., Croy B. A., Marshall J. S., Chaouat G., Clark D. A. (1997). Stress triggered abortions are associated with alterations of granulated cells into the decidua.. Am J Reprod Immunol.

[PDF_01400] Mattsson R., Holmdahl R., Scheynius A., Bernadotte F., Mattsson A., Van der Meide P. H. (1991). Placental MHC class I antigen expression is induced in mice following in vivo treatment with recombinant interferon-gamma.. J Reprod Immunol.

[PDF_01684] Mattsson R., Holmdahl R., Scheynius A., Bernadotte F., Mattsson A., Van der Meide P. H. (1991). Placental MHC class I antigen expression is induced in mice following in vivo treatment with recombinant interferon-gamma.. J Reprod Immunol.

[PDF_01252] Mattsson R., Mattsson A., Sulila P. (1985). Allogeneic pregnancy in B-lymphocyte deprived CBA/Ca mice--effects on maternal lymphoid organs and fetal survival.. Dev Comp Immunol.

[PDF_01961] Menu E., Jankovic D. L., Theze J., David V., Chaouat G. (1990). Immunoactive products of human placenta (III): Characterization of an inhibitor affecting lymphocyte proliferation.. Reg Immunol.

[PDF_01956] Menu E., Kaplan L., Andreu G., Denver L., Chaouat G. (1989). Immunoactive products of human placenta. I. An immunoregulatory factor obtained from explant cultures of human placenta inhibits CTL generation and cytotoxic effector activity.. Cell Immunol.

[PDF_01965] Menu E., Kinsky R., Hoffman M., Chaouat G. (1991). Immunoactive products of human placenta. IV. Immunoregulatory factors obtained from cultures of human placenta inhibit in vivo local and systemic allogeneic and graft versus-host reactions in mice.. J Reprod Immunol.

[PDF_01991] Migita K., Eguchi K., Kawabe Y., Tsukada T., Ichinose Y., Nagataki S., Ochi A. (1995). Defective TCR-mediated signaling in anergic T cells.. J Immunol.

[PDF_01995] Mincheva-Nilsson L., Hammarström S., Hammarström M. L. (1992). Human decidual leukocytes from early pregnancy contain high numbers of gamma delta+ cells and show selective down-regulation of alloreactivity.. J Immunol.

[PDF_01915] Mowbray J., Jalali R., Chaouat G., Clark D. A., Underwood J., Allen W. R., Mathias S. (1997). Maternal response to paternal trophoblast antigens.. Am J Reprod Immunol.

[PDF_01692] PARANT M., CHEDID L. (1964). PROTECTIVE EFFECT OF CHLORPROMAZINE AGAINST ENDOTOXIN-INDUCED ABORTION.. Proc Soc Exp Biol Med.

[PDF_01620] Pampfer S., Tabibzadeh S., Chuan F. C., Pollard J. W. (1991). Expression of colony-stimulating factor-1 (CSF-1) messenger RNA in human endometrial glands during the menstrual cycle: molecular cloning of a novel transcript that predicts a cell surface form of CSF-1.. Mol Endocrinol.

[PDF_01695] Poff J. P., Fairchild D. L., Condon W. A. (1988). Effects of antibiotics and medium supplements on steroidogenesis in cultured cow luteal cells.. J Reprod Fertil.

[PDF_01448] Pollard J. W., Hunt J. S., Wiktor-Jedrzejczak W., Stanley E. R. (1991). A pregnancy defect in the osteopetrotic (op/op) mouse demonstrates the requirement for CSF-1 in female fertility.. Dev Biol.

[PDF_01656] Roberts R. M., Cross J. C., Leaman D. W. (1992). Interferons as hormones of pregnancy.. Endocr Rev.

[PDF_01482] Robertson S. A., Mau V. J., Hudson S. N., Tremellen K. P. (1997). Cytokine-leukocyte networks and the establishment of pregnancy.. Am J Reprod Immunol.

[PDF_01473] Robertson S. A., Seamark R. F., Guilbert L. J., Wegmann T. G. (1994). The role of cytokines in gestation.. Crit Rev Immunol.

[PDF_01248] Rodger J. C. (1985). Lack of a requirement for a maternal humoral immune response to establish or maintain successful allogeneic pregnancy.. Transplantation.

[PDF_01297] Romero R., Mazor M., Tartakovsky B. (1991). Systemic administration of interleukin-1 induces preterm parturition in mice.. Am J Obstet Gynecol.

[PDF_01741] Roth I., Corry D. B., Locksley R. M., Abrams J. S., Litton M. J., Fisher S. J. (1996). Human placental cytotrophoblasts produce the immunosuppressive cytokine interleukin 10.. J Exp Med.

[PDF_01944] Saji F., Koyama M., Kameda T., Negoro T., Nakamuro K., Tanizawa O. (1987). Effect of a soluble factor secreted from cultured human trophoblast cells on in vitro lymphocyte reactions.. Am J Reprod Immunol Microbiol.

[PDF_01438] Sanford T. R., De M., Wood G. W. (1992). Expression of colony-stimulating factors and inflammatory cytokines in the uterus of CD1 mice during days 1 to 3 of pregnancy.. J Reprod Fertil.

[PDF_01530] Seifer D. B., Romero R., Berlinsky D., Haning R. V. (1993). Absence of immunoreactive cytokines in supernatants of individual preimplantation human embryos.. Am J Reprod Immunol.

[PDF_01525] Sheth K. V., Roca G. L., al-Sedairy S. T., Parhar R. S., Hamilton C. J., al-Abdul Jabbar F. (1991). Prediction of successful embryo implantation by measuring interleukin-1-alpha and immunosuppressive factor(s) in preimplantation embryo culture fluid.. Fertil Steril.

[PDF_01502] Simón C., Frances A., Lee B. Y., Mercader A., Huynh T., Remohi J., Polan M. L., Pellicer A. (1995). Immunohistochemical localization, identification and regulation of the interleukin-1 receptor antagonist in the human endometrium.. Hum Reprod.

[PDF_01485] Simón C., Frances A., Piquette G. N., el Danasouri I., Zurawski G., Dang W., Polan M. L. (1994). Embryonic implantation in mice is blocked by interleukin-1 receptor antagonist.. Endocrinology.

[PDF_01506] Simón C., Frances A., Piquette G., Hendrickson M., Milki A., Polan M. L. (1994). Interleukin-1 system in the materno-trophoblast unit in human implantation: immunohistochemical evidence for autocrine/paracrine function.. J Clin Endocrinol Metab.

[PDF_01533] Simón C., Mercader A., Gimeno M. J., Pellicer A. (1997). The interleukin-1 system and human implantation.. Am J Reprod Immunol.

[PDF_01364] Soubiran P., Zapitelli J. P., Schaffar L. (1987). IL2-like material is present in human placenta and amnion.. J Reprod Immunol.

[PDF_01516] Steele G. L., Currie W. D., Leung E. H., Yuen B. H., Leung P. C. (1992). Rapid stimulation of human chorionic gonadotropin secretion by interleukin-1 beta from perifused first trimester trophoblast.. J Clin Endocrinol Metab.

[PDF_01367] Stern P. L., Beresford N., Friedman C. I., Stevens V. C., Risk J. M., Johnson P. M. (1987). Class I-like MHC molecules expressed by baboon placental syncytiotrophoblast.. J Immunol.

[PDF_01488] Stewart C. L., Kaspar P., Brunet L. J., Bhatt H., Gadi I., Köntgen F., Abbondanzo S. J. (1992). Blastocyst implantation depends on maternal expression of leukaemia inhibitory factor.. Nature.

[PDF_01865] Stoppacciaro A., Melani C., Parenza M., Mastracchio A., Bassi C., Baroni C., Parmiani G., Colombo M. P. (1993). Regression of an established tumor genetically modified to release granulocyte colony-stimulating factor requires granulocyte-T cell cooperation and T cell-produced interferon gamma.. J Exp Med.

[PDF_01672] Sunder-Plassmann G., Derfler K., Wagner L., Stockenhuber F., Endler M., Nowotny C., Balcke P. (1989). Increased serum activity of interleukin-2 in patients with pre-eclampsia.. J Autoimmun.

[PDF_01714] Szekeres-Bartho J., Faust Z., Varga P., Szereday L., Kelemen K. (1996). The immunological pregnancy protective effect of progesterone is manifested via controlling cytokine production.. Am J Reprod Immunol.

[PDF_01711] Szekeres-Bartho J., Wegmann T. G. (1996). A progesterone-dependent immunomodulatory protein alters the Th1/Th2 balance.. J Reprod Immunol.

[PDF_01327] Tafuri A., Alferink J., Möller P., Hämmerling G. J., Arnold B. (1995). T cell awareness of paternal alloantigens during pregnancy.. Science.

[PDF_01773] Tangri S., Raghupathy R. (1993). Expression of cytokines in placentas of mice undergoing immunologically mediated spontaneous fetal resorptions.. Biol Reprod.

[PDF_01778] Tangri S., Wegmann T. G., Lin H., Raghupathy R. (1994). Maternal anti-placental reactivity in natural, immunologically-mediated fetal resorptions.. J Immunol.

[PDF_01580] Taupin J. L., Acres B., Dott K., Schmitt D., Kieny M. P., Gualde N., Moreau J. F. (1993). Immunogenicity of HILDA/LIF either in a soluble or in a membrane anchored form expressed in vivo by recombinant vaccinia viruses.. Scand J Immunol.

[PDF_01336] Tranchot-Diallo J., Gras G., Parnet-Mathieu F., Benveniste O., Marcé D., Roques P., Milliez J., Chaouat G., Dormont D. (1997). Modulations of cytokine expression in pregnant women.. Am J Reprod Immunol.

[PDF_01266] Wegmann T. G. (1984). Foetal protection against abortion: is it immunosuppression or immunostimulation?. Ann Immunol (Paris).

[PDF_01730] Wegmann T. G., Lin H., Guilbert L., Mosmann T. R. (1993). Bidirectional cytokine interactions in the maternal-fetal relationship: is successful pregnancy a TH2 phenomenon?. Immunol Today.

[PDF_01390] Wegmann T. G., Singh B., Carlson G. A. (1979). Allogeneic placenta is a paternal strain antigen immunoabsorbent.. J Immunol.

[PDF_01664] Whaley A. E., Meka C. S., Harbison L. A., Hunt J. S., Imakawa K. (1994). Identification and cellular localization of unique interferon mRNA from human placenta.. J Biol Chem.

[PDF_01613] Wu M. C., Yunis A. A. (1980). Common pattern of two distinct types of colony-stimulating factor in human tissues and cultured cells.. J Clin Invest.

[PDF_01599] Yang Y., Yelavarthi K. K., Chen H. L., Pace J. L., Terranova P. F., Hunt J. S. (1993). Molecular, biochemical, and functional characteristics of tumor necrosis factor-alpha produced by human placental cytotrophoblastic cells.. J Immunol.

[PDF_01594] Zhou Y., Damsky C. H., Chiu K., Roberts J. M., Fisher S. J. (1993). Preeclampsia is associated with abnormal expression of adhesion molecules by invasive cytotrophoblasts.. J Clin Invest.

[PDF_01414] Zuckermann F. A., Head J. R. (1987). Murine trophoblast resists cell-mediated lysis. I. Resistance to allospecific cytotoxic T lymphocytes.. J Immunol.

[PDF_01417] Zuckermann F. A., Head J. R. (1988). Murine trophoblast resists cell-mediated lysis. II. Resistance to natural cell-mediated cytotoxicity.. Cell Immunol.

[PDF_01840] de Fougerolles A. R., Baines M. G. (1987). Modulation of the natural killer cell activity in pregnant mice alters the spontaneous abortion rate.. J Reprod Immunol.

[PDF_01350] van der Ven K., Ober C. (1994). HLA-G polymorphisms in African Americans.. J Immunol.

